# Sex Differences in 6-Year Change in eGFR and ACR in a Multiethnic Population: The HELIUS Study

**DOI:** 10.1016/j.xkme.2026.101381

**Published:** 2026-04-30

**Authors:** Taryn G. Vosters, Muhulo M. Mungamba, Sarah A. van Eeghen, Vianda S. Stel, Felix P. Chilunga, Daniel H. van Raalte, Kitty J. Jager, Bert-Jan H. van den Born, Frans J. van Ittersum, Liffert Vogt, Irene G.M. van Valkengoed

**Affiliations:** 1 Department of Public and Occupational Health, Amsterdam UMC, University of Amsterdam, Amsterdam, the Netherlands; 2 Center of Expertise on Gender Dysphoria, Department of Internal Medicine, Amsterdam University Medical Center, Amsterdam, The Netherlands; 3 Amsterdam Gastroenterology Endocrinology Metabolism, Amsterdam UMC, Amsterdam, The Netherlands; 4 Department of Endocrinology and Metabolism, Amsterdam UMC, Amsterdam, The Netherlands; 5 Department of Medical Informatics, Amsterdam UMC, Location AMC, Amsterdam, the Netherlands; 6 Diabetes Centre, Department of Internal Medicine, Amsterdam University Medical Centre, Amsterdam, The Netherlands; 7 Amsterdam Cardiovascular Sciences, Vrije Universiteit, Amsterdam, The Netherlands; 8 Department of Internal Medicine, Section Nephrology, Amsterdam UMC, Vrije Universiteit Amsterdam, Amsterdam, the Netherlands; 9 Department of Internal Medicine, Section Nephrology, Amsterdam UMC, University of Amsterdam, Amsterdam, the Netherlands

**Keywords:** CKD progression, kidney function decline, sex differences

## Abstract

**Rationale & Objective:**

Data on sex differences in kidney function within multiethnic populations are scarce despite the large differences in chronic kidney disease (CKD) in women and men. We investigated the 6-year changes in the estimated glomerular filtration rate (eGFR) and albumin-creatinine ratio (ACR) in women and men, both overall and across ethnic groups, and determined whether sex differences in associations with outcomes are mediated by known CKD risk factors.

**Study Design:**

General population based longitudinal study.

**Setting & Participants:**

We used prospective data of 5,713 women and 4,407 men from 6 ethnic backgrounds from the Healthy Life in an Urban Setting (HELIUS) study (Amsterdam, the Netherlands).

**Exposures:**

Municipality registered sex.

**Outcomes:**

Change in eGFR (mL/min/1.73 m^2^) and ACR (mg/mmol) between baseline (2011-2015) and follow-up (2019-2021) data collection. CKD incidence and progression were analyzed as secondary outcomes.

**Analytical Approach:**

Linear regression analyses adjusted for baseline kidney function estimates, follow-up duration, age, education, and ethnicity in the total population and stratified by ethnicity. Mediation by hypertension, diabetes, cardiovascular disease, obesity, physical activity, alcohol consumption, and smoking was tested.

**Results:**

Although no significant sex differences were found in the overall population, Dutch and African Surinamese women had a greater decrease in eGFR (β:1.2 (0.5-1.8)) than men, whereas South-Asian Surinamese and Moroccan men had a greater decrease in eGFR (β:–1.0 (–2.0 to 0.0)) than women. ACR was higher in men, although this difference did not reach statistical significance (β:0.6 (0.3-1.1), *P* = 0.09). Sex differences in CKD incidence across certain ethnic groups aligned with the observed eGFR differences, whereas CKD progression was higher in men overall. Little evidence of mediation by CKD risk factors of the sex differences was observed.

**Limitations:**

Single eGFR and ACR measurements, self-reported mediator variables, and limited generalizability.

**Conclusions:**

Disparate sex differences in the changes in eGFR, CKD incidence, and progression were observed, specifically in some ethnic groups. These differences were not mediated by differences in traditional risk factors and health-related behaviors.

## Plain-language Summary

The study investigated whether kidney function decline differed between women and men in a multiethnic population. We further investigated whether the sex differences in the overall group were evident across the included 6 ethnic groups. No sex differences were found in the total population; however, we observed sex differences in some ethnic groups. These differences were not explained by the traditional CKD risk factors. Possible explanations for the sex differences in ethnic groups may include disparities in sex hormones, CKD risk factors, and possible chance variations because of differing sample sizes. Future studies should continue including diverse populations to potentially discover the underlying mechanisms behind the variations in sex differences across ethnic groups.

Chronic kidney disease (CKD) is a major public health concern and is estimated to affect approximately 800 million people worldwide.^1^ CKD is a progressive disease, which can lead to premature death and kidney failure.^2^ Despite its widespread occurrence, CKD, classified as an estimated glomerular filtration rate (eGFR) <60 mL/min/1.73 m^2^ and/or albumin-creatinine ratio (ACR) ≥3 mg/mmol over a minimum of 3 months, exhibits distinct patterns across demographic groups, eg, in women versus men and across ethnic groups.^3^^,^^4^ To identify population groups who should be targeted for prevention and treatment, we need to study the changes in eGFR and ACR that contribute to the incidence and progression of CKD across sex- and ethnicity-diverse population groups.

Although kidney function declines naturally with age in all individuals, CKD incidence rates are consistently reported to be higher in women.^5^^,^^6^ This difference may be partially attributed to hormonal factors, with estrogen playing a protective role in kidney function in premenopausal women.^7^, ^8^, ^9^ However, this protection diminishes after menopause, potentially leading to a faster decline in kidney function because of reduced estrogen levels. Anatomical differences, such as smaller glomeruli in women, might also contribute to CKD development, alongside socioeconomic factors such as limited access to health care in certain groups.^8^ However, several studies have suggested that men are more likely to experience faster CKD progression than women.^3^^,^^6^ Evidence on sex differences in kidney function decline and CKD progression is been universally inconsistent in both the general and patient populations.^9^, ^10^, ^11^, ^12^, ^13^, ^14^ For example, Araumi et al^13^ (2021) showed that in a healthy Japanese population, the change in eGFR was slightly higher in women but very similar to that in men after a 1-year follow-up, whereas the Dutch population-based PREVEND (Prevention of Renal and Vascular Endstage Disease) study found that men had a higher rate of eGFR decline than women.^12^ Whether these sex disparities extend to a multiethnic population, such as that included in the Healthy Life in an Urban Setting (HELIUS) study, remains unclear. Addressing this gap in knowledge is important given the potential disparities in disease severity and outcomes across different population groups.^15^

Several studies have investigated CKD progression across different ethnic groups in the United States and the United Kingdom.^16^, ^17^, ^18^ A diverse community study in the United States found that African-American and Hispanic groups without cardiovascular disease experienced elevated rates of kidney function decline compared with the European-origin group, whereas Chinese groups exhibited similar rates of decline to the European groups.^19^ Data on sex differences in multi-ethnic groups and from regions aside from the United States and the United Kingdom are scarce.^20^ Expanding the study of CKD in women and men across other populations outside of the United States and the United Kingdom is crucial for understanding the global variability in kidney function decline and CKD progression and ensuring that the findings are applicable to diverse populations, particularly in regions with rising CKD rates. The HELIUS study provides a unique context to do so, including data on women and men from six ethnic groups aged 18-70 years living in Amsterdam, the Netherlands.

This study used data from HELIUS to explore whether disparities are present in the change in eGFR, ACR, and secondary outcomes (CKD incidence and progression) over time in women and men, both overall and across six ethnic groups. We assessed whether any sex differences persisted when socio-cultural factors such as education were adjusted for. Second, we investigated whether sex disparities were mediated by traditional CKD risk factors (hypertension, diabetes, and cardiovascular disease) and factors reflecting health-related behaviors, such as obesity, physical activity, alcohol consumption, and smoking (Fig S1).

## Materials and Methods

### Data and Study Population

This study made use of the longitudinal data from the multiethnic Healthy Living In Urban Setting (HELIUS) Study conducted on participants living in Amsterdam, the Netherlands. A detailed description of this study has been published elsewhere.^21^^,^^22^ Participants aged 18-70 years old with a municipality-registered Dutch, Surinamese, Ghanaian, Turkish, or Moroccan ethnicity were invited to participate in the study. The invitations were sent via mail or home visits. If necessary, language interpreters visited their homes. Baseline data, including a questionnaire and biological samples (fasting blood and urine), were collected between 2011 and 2015. All participants who took part in the baseline data collection were invited to participate in follow-up data collection. The HELIUS study had a retention rate of 46% at the 6-year follow-up, which varied between ethnic groups, with higher retention in the Dutch and Surinamese groups than in the other groups.^22^ The second wave of data collection was conducted between May 2019 and November 2022; however, for organizational reasons collection of urine samples, as part of the standard measurement protocol, was restricted to participants who visited between June 2021 and November 2022. Participants who participated before the start of urine collection were invited back to donate their urine samples and an additional nonfasting serum sample for the current study. This resulted in the follow-up ACR group being smaller than that of the serum creatinine (eGFR) group. Ethical approval was obtained from the Ethical Review Board of the Academic Medical Center Amsterdam (MREC 10/100# 17.10.1729), and all participants provided written informed consent. The study adhered to the principles stated in the declaration of Helsinki.

The current study included all participants who completed questionnaires, physical examination, and blood and/or urine sample collection at baseline and follow-up (n = 10,120). Participants with Javanese, other/unknown Surinamese, or other/unknown ethnic backgrounds were excluded from the study because of small sample sizes (n = 548). Cases with no available data on any CKD outcomes (ie, eGFR and ACR; n = 193) and all traditional risk factors (n = 145) at baseline and follow-up were excluded from the study. The final cohort consisted of 4,407 participants registered as men and 5,713 participants registered as women, of whom 7,536 participants were included in the ACR subset.

### Data

All variable definitions are presented in Table 1.^4^^,^^23^

**Table 1:** Variable Descriptions

Variable	Measurement	Definition
Kidney Function Estimates		
Change in eGFR	At both baseline and follow-up, the eGFR was estimated using the CKD Epidemiology (CKD-EPI) 2021 equation using serum creatinine obtained from a single fasting venous blood sample. Plasma creatinine concentration was determined using a kinetic colorimetric spectrophotometric enzyme assay (Roche Diagnostics, Japan). Cystatin C levels were not available at the time of the study.	Changes in eGFR were calculated by subtracting baseline estimates from follow-up estimates.
Change in ACR	Albuminuria was assessed by measuring the ACR in a single first morning urine sample. Kinetic spectrophotometric and immune chemical turbidimetric methods were used to analyze the urine creatinine and albumin concentrations.	Changes in ACR were calculated by subtracting baseline estimates from follow-up estimates.
CKD prevalence	See measurement of eGFR and ACR for measurements specifications.	CKD prevalence was defined as eGFR < 60 mL/min/1.73 m^2^ and/or ACR ≥ 3 mg/mmol at baseline, according to the KDIGO guidelines.^4^
CKD incidence	See measurement of eGFR and ACR for measurement specifications.	CKD incidence was defined as eGFR < 60 mL/min/1.73 m^2^ and/or ACR ≥ 3mg/mmol at follow-up with no CKD diagnosis at baseline, according to the KDIGO guidelines.^4^
CKD progression	See measurement of eGFR and ACR for measurement specifications.	CKD progression was defined as a drop in CKD stage or a 25% decline from the baseline estimate in participants with CKD per the KDIGO guideline states.^4^
Other Variables		
Sex	Sex was registered with the municipality.	Categorized as woman or man.
Age	Age was registered with the municipality.	Years (continuous).
Ethnicity	Based on the registered country of birth of the participant and parent and via a questionnaire. Surinamese participants were further categorized into subgroups South-Asian or African based on self-reported identity.	Participants were considered non-Dutch if they and one parent were born outside the Netherlands or if they were born in the Netherlands, and both parents were born outside the Netherlands.
Educational level	Determined using a questionnaire based on the highest attained educational level, either in or outside the Netherlands.	This was further categorized into “low” (elementary school or lower), “low-medium” (lower secondary schooling), “medium-high” (higher secondary schooling), and “high” (higher vocational schooling or university).
Duration from baseline to follow-up	The difference between date of baseline data collection and follow-up date of data collection.	Months (continuous).
Hypertension	BP was measured twice on the left arm in a seated position after 5 min of rest.	Defined as a mean diastolic BP ≥ 90 mm Hg, and/or systolic BP ≥ 140 mm Hg, and/or the use of antihypertensive medications (angiotensin-converting enzyme inhibitors, angiotensin II receptor blockers, diuretics, or β-blockers) at the time of collection.
Diabetes mellitus	Fasting blood samples provided a single fasting glucose measurement.	Defined as fasting glucose ≥7 mmol/L, self-reported diagnosis of diabetes, or use of glucose lowering medication.
Cardiovascular disease	Participants stated in their questionnaire ever having had the following: stroke/heart attack/bypass or stent surgery.	Classified if participants stated having one or more of the following stroke/heart attack/bypass or stent surgery.
Obesity	Based on the body mass index measured at time of collection.	Categorized as “normal” < 25 kg/m^2^; “overweight” 25≤30 kg/m^2^; “obese” >30 kg/m^2^.
Physical activity	Assessed using the SQUASH Questionnaire.^23^	Meeting the recommendation given by Dutch guidelines of >30 min for 5 days per week: “yes” or “no”.
Alcohol consumption	Self-reported using a questionnaire.	categorized as none, low, moderate, or high over the past 12 months
Smoking status	Self-reported using a questionnaire.	Smoking status was categorized as “current smoker” or “non-smoker/former smoker”.

Abbreviations: BP, Blood pressure; SQUASH, Short Questionnaire to Assess Health-Enhancing Physical Activity.

### Statistical Analyses

The baseline characteristics of women and men in the total population and across ethnic groups were described using percentages and frequencies for categorical variables, means (SD) for normally distributed data, and median (IQR) for non-normal distributions (evaluated via visual inspection of frequency and QQ plots). Characteristics of the subset with an ACR measurement and of the total population at the 6-year follow-up are provided as a supplement.

Differences in eGFR (estimated using the Chronic Kidney Disease Epidemiology Collaboration [CKD-EPI] 2021 formula) and ACR and secondary measures of CKD incidence and progression were also described in the total population and across ethnic groups. We then determined the association between sex and changes in eGFR and ACR, adjusted for follow-up duration and baseline eGFR and ACR values (model 1) with linear regression analyses in the total population. To further explore this relationship, we additionally adjusted for age, ethnicity, and education (model 2, main model). We repeated all the analyses stratified by ethnicity. An interaction term between sex and ethnicity was added to the models in the total population to formally assess the consistency of the patterns of sex differences across ethnic groups.

Lastly, a mediation analysis using the R package *mediation* to assess direct and indirect effects explored the extent to which associations observed between sex and changes in our primary outcomes (eGFR and ACR) over time were explained by baseline alcohol consumption, physical activity, obesity, smoking, hypertension, diabetes, and cardiovascular disease (CVD).^24^ Additionally, in a supplement, we conducted mediation analyses according to the Baron and Kenny method to confirm consistency in our results, independent of the method employed.

In addition, in sensitivity analyses within the group without CKD at baseline, we studied sex differences in CKD incidence as well as sex differences in progression in the group with CKD. For these analyses, we used logistic regression with adjustments similar to the main analyses. Moreover, sensitivity analyses investigated the relationship between sex and CKD progressors (individuals with significant CKD progression) and nonprogressors (those that did not have significant CKD progression).

Finally, in post hoc analyses, we explored whether attrition potentially influenced our findings. We repeated the analyses of the crude changes in the primary outcomes and of the main model with the primary outcomes using inverse probability weighting (IPW) to account for missing data at follow-up. Lastly, we divided the groups into those below and above 50 years of age to explore whether sex differences varied between age groups.

All *P* values were considered significant at *P* < 0.05, and all analyses were performed using R Studio 4.2.1.

## Results

The overall mean age for women was 46.1 (SD 12.3) years and 46.5 (SD 12.5) years for men (Table 2). Low education was more prevalent in women than in men (15.6% vs 9.4%). Men had a higher prevalence of diabetes, hypertension, CVD, and smoking than women, whereas women had higher proportions of obesity, high alcohol consumption, and nonadherence to physical activity guidelines. These patterns of sex differences varied somewhat across ethnic groups. For instance, Dutch women had lower rates of obesity than Dutch men, and African Surinamese women had higher rates of CVD than African Surinamese men. Patterns of differences in characteristics were similar in the subset with an ACR measurement as well as at the 6-year follow-up (Tables S1-S3).

**Table 2:** Baseline Characteristics of Women and Men in the Total Population and by Ethnicity

		Women							Men						
		Overall (n = 5,713)	Dutch (n = 1,548)	SA Surinamese (n = 962)	African Surinamese (n = 1,315)	Ghanaian (n = 478)	Turkish (n = 547)	Moroccan (n = 863)	Overall (n = 4,407)	Dutch (n = 1401)	SA Surinamese (n = 734)	African Surinamese (n = 759)	Ghanaian (n = 324)	Turkish (n = 498)	Moroccan (n = 691)
Age (y)		46.1 (12.3)	47.4 (13.1)	47.3 (12.1)	49.3 (11.4)	44.1 (9.5)	41.5 (11.5)	41.7 (12.0)	46.5 (12.5)	48.0 (13.0)	46.0 (12.9)	49.6 (11.6)	48.1 (10.4)	41.4 (11.5)	43.4 (11.9)
Educational level	Low	886 (15.6)	38 (2.5)	82 (11.2)	55 (4.2)	170 (36.2)	183 (33.9)	305 (35.5)	435 (9.94)	36 (2.6)	82 (11.2)	33 (4.4)	50 (15.6)	84 (17.0)	150 (21.9)
	Low-Medium	1,337 (23.5)	198 (12.8)	333 (34.8)	390 (29.8)	175 (37.2)	96 (17.8)	145 (16.9)	1117 (25.5)	162 (11.6)	218 (29.8)	296 (39.4)	142 (44.4)	148 (30.0)	151 (22.0)
	Medium high	1,599 (28.2)	318 (20.6)	280 (29.3)	487 (37.2)	103 (21.9)	154 (28.5)	257 (29.9)	1,254 (28.7)	315 (22.6)	216 (29.5)	249 (33.2)	94 (29.4)	150 (30.4)	230 (33.6)
	High	1,854 (32.6)	988 (64.1)	208 (21.8)	376 (28.7)	22 (4.7)	107 (19.8)	153 (17.8)	1,567 (35.8)	879 (63.1)	216 (29.5)	173 (23.0)	34 (10.6)	111 (22.5)	154 (22.5)
Alcohol consumption	Low	4,236 (74.5)	504 (32.7)	839 (87.8)	1121 (85.8)	421 (89.4)	508 (92.9)	843 (98.0)	3,052 (69.6)	530 (37.9)	581 (79.5)	563 (74.9)	277 (86.6)	447 (90.3)	654 (95.1)
	Moderate	956 (16.8)	641 (41.6)	91 (9.5)	141 (10.8)	40 (8.5)	30 (5.5)	13 (1.5)	1,023 (23.3)	680 (48.6)	102 (13.9)	148 (19.7)	42 (13.1)	30 (6.1)	21 (3.0)
	High	491 (8.6)	397 (25.7)	26 (2.7)	44 (3.4)	10 (2.1)	9 (1.6)	5 (0.6)	310 (7.1)	189 (13.5)	48 (6.6)	41 (5.5)	1 (0.3)	18 (3.6)	13 (1.9)
Physical activity^a^	Yes	3,282 (57.5)	1219 (78.8)	485 (50.05)	763 (58.0)	238 (49.9)	211 (38.6)	366 (42.4)	2,826 (64.2)	1021 (78.8)	435 (59.3)	521 (68.7)	211 (65.1)	251 (50.5)	387 (56.2)
	No	2,426 (42.5)	328 (21.2)	475 (49.5)	552 (42.0)	239 (50.10)	335 (61.4)	497 (57.6)	1,575 (35.8)	379 (27.1)	298 (40.7)	237 (31.3)	113 (34.9)	246 (49.5)	302 (43.8)
Obesity	Yes	1,485 (26.0)	129 (8.3)	212 (22.0)	467 (35.6)	206 (43.2)	178 (32.5)	293 (34.0)	620 (14.1)	131 (9.4)	82 (11.2)	108 (14.2)	52 (16.1)	122 (24.5)	125 (18.1)
	No	4,224 (74.0)	1,418 (91.7)	750 (78.0)	846 (64.4)	271 (56.8)	369 (67.5)	570 (66.0)	3,785 (85.9)	1270 (90.6)	651 (88.8)	651 (85.8)	271 (83.9)	376 (75.5)	566 (81.9)
Smoking	Current	946 (16.6)	320 (20.7)	163 (17.0)	271 (20.7)	9 (1.9)	147 (27.0)	36 (4.2)	1,171 (26.6)	326 (23.3)	235 (32.1)	297 (39.4)	30 (9.3)	159 (32.2)	124 (18.0)
	Former/Never	4746 (83.4)	1,225 (79.3)	794 (83.0)	1038 (79.3)	467 (98.1)	397 (73.0)	825 (95.8)	3,220 (73.3)	1,074 (76.7)	497 (67.9)	457 (60.6)	292 (90.7)	335 (67.8)	565 (82.0)
Hypertension	Yes	1,663 (29.1)	277 (17.9)	316 (32.8)	601 (45.7)	228 (47.7)	99 (18.1)	142 (16.5)	1,600 (36.3)	454 (32.4)	300 (40.9)	355 (46.8)	185 (57.1)	140 (28.1)	166 (24.0)
	No	4,050 (70.9)	1,271 (82.1)	646 (67.2)	714 (54.3)	250 (52.3)	448 (81.9)	721 (83.5)	2807 (63.7)	947 (32.4)	434 (59.1)	404 (53.2)	139 (42.9)	358 (71.9)	525 (76.0)
Diabetes	Yes	485 (8.5)	32 (2.1)	148 (15.4)	134 (10.2)	45 (9.4)	38 (6.9)	88 (10.2)	451 (10.2)	63 (4.5)	158 (21.5)	73 (9.6)	38 (11.7)	43 (8.6)	76 (11.0)
	No	5,228 (91.5)	1,516 (97.9)	814 (84.6)	1,181 (89.8)	433 (90.6)	509 (93.1)	775 (89.8)	3,956 (89.8)	1,338 (95.5)	576 (78.5)	686 (90.4)	286 (88.3)	455 (91.4)	615 (89.0)
CVD	Yes	756 (13.2)	90 (5.8)	157 (16.3)	196 (14.9)	70 (14.6)	104 (19.0)	139 (16.1)	603 (13.7)	118 (8.4)	156 (21.3)	103 (13.6)	42 (13.0)	83 (16.7)	101 (14.6)
	No	4957 (86.8)	1,458 (94.2)	805 (83.7)	1,119 (85.1)	408 (85.4)	443 (81.0)	724 (83.9)	3,804 (86.3)	1,283 (91.6)	578 (78.7)	656 (86.4)	282 (87.0)	415 (83.3)	590 (85.4)

Abbreviation: SA Surinamese, South-Asian Surinamese.

a Defined by adherence to Dutch physical activity guidelines.

Compared with participants included in the final study population, participants lost to follow-up had higher prevalences of low educational status, as well as higher hypertension, obesity, and diabetes prevalences (Table S4).

### Kidney Function Outcomes

**Figure 1:**
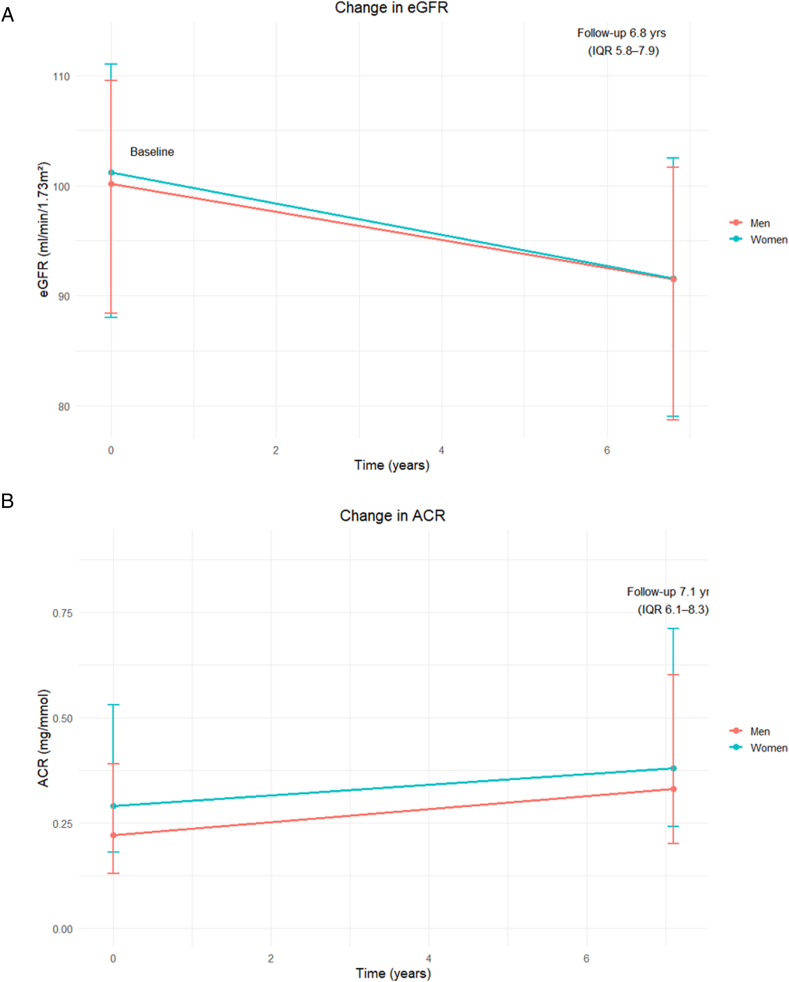
(A) Estimated glomerular filtration rate and (B) albumin-creatinine ratio at baseline and 6-year follow-up in women and men in the overall population. Graph reflects 2 measurements at two time points, however it is not intended to represent the process of decline. Abbreviations: ACR, albumin-creatinine ratio estimated in mg/mmol; Afr. Surinamese, African Surinamese; dashed line, men; dotted line, women; eGFR, estimated glomerular filtration rate (mL/min/1.73 m^2^) using the CKD-EPI 2021 equation; SA Surinamese, South-Asian Surinamese.

Women and men had similar overall median eGFR estimates at baseline and at the 6-year follow-up (eg, baseline: 101.19 (88.01-111.05) mL/min/1.73 m^2^ in women vs 100.20 (88.43-109.67) mL/min/1.73 m^2^ in men). Crude change over time was also similar in women and men (–8.12 (–13.88 to –4.05) mL/min/1.73 m^2^ in women vs –7.87 (–13.95 to –3.87) mL/min/1.73 m^2^ in men; Table 3, Figure 1). However, variations were observed across ethnic groups, including a larger crude eGFR change over time estimates in Dutch, African Surinamese, and Ghanaian women than in men, suggesting a faster eGFR decline in women, and the reverse was noted for South-Asian Surinamese, Turkish, and Moroccan women compared with men. The median baseline and 6-year follow-up ACR estimates were higher in women than in men, both overall and across ethnic groups, aside from the Turkish group (Fig S2). Contrastingly, men had a larger crude change in ACR than women (0.07 (0.10-0.30) mg/mmol in women versus 0.09 (–0.03 to 0.30) mg/mmol in men), suggesting a larger increase in ACR concentrations in men. The estimates and patterns of change in eGFR and ACR were similar in the IPW analyses (Table S5).

**Table 3:** Chronic Kidney Disease-Related Outcomes at Baseline and After 6-Year Follow-up in Women and Men in the Total Population and by Ethnicity

		Women							Men						
		Overall	Dutch	SA Surinamese	Afr. Surinamese	Ghanaian	Turkish	Moroccan	Overall	Dutch	SA Surinamese	Afr. Surinamese	Ghanaian	Turkish	Moroccan
Median eGFR [mL/min/1.73 m^2^]	Baseline	101.19 (88.01 to 111.05)	96.34 (84.82 to105.11)	103.04 (91.94 to 110.70)	92.63 (80.97 to 103.74)	95.85 (84.38 to106.79)	110.80 (104.33 to118.89)	113.37 (105.86 to 120.65)	100.20 (88.43 to 109.67)	100.26 (89.97 to 109.53)	98.47 (87.96 to 107.76)	89.66 (79.58 to 100.92)	87.81 (77.15 to 98.58)	108.25 (100.19 to 116.74)	108.00 (100.63-116.04)
	Follow-up	91.57 (79.04 to 102.51)	87.98 (77.13 to97.48)	92.17 (81.05 to 101.13)	82.39 (72.42 to 93.98)	87.72 (77.05 to 97.46)	103.07 (93.92 to 109.76)	105.05 (95.69 to 113.36)	91.52 (78.73 to 101.66)	93.05 (81.56 to 101.66)	88.47 (76.86 to 99.22)	82.18 (71.61 to 93.29)	79.26 (69.54 to 91.74)	100.26 (89.42 to 107.46)	98.15 (89.02-106.24)
	Change	–8.12 (–13.88 to –4.05)	–7.81 (–13.57 to –2.82)	–9.04 (–15.49 to –4.87)	–9.03 (–14.74 to –3.72)	–7.48 (–13.15 to –3.08)	–7.84 (–12.54 to –4.88)	–7.73 (–12.14 to –5.17)	–7.87 (–13.95 to –3.87)	–6.94 (–12.26 to –3.15)	–9.87 (–16.63 to –4.58)	–6.84 (–13.5 to –2.07)	–7.05 (–13.21 to –1.39)	8.29 (–14.13 to –5.00)	–9.04 (–14.22 to –5.98)
Median ACR [mg/mmol]^a^	Baseline	0.29 (0.18 to 0.53)	0.26 (0.18 to 0.42)	0.30 (0.19 to0.58)	0.28 (0.17 to 0.54)	0.28 (0.18 to0.58)	0.35 (0.21-0.63)	0.36 (0.22 to 0.76)	0.22 (0.13 to 0.39)	0.22 (0.15 to 0.36)	0.23 (0.13 to 0.45)	0.19 (0.12 to 0.35)	0.19 (0.11 to 0.41)	0.24 (0.15-0.42)	0.25 (0.15 to 0.49)
	Follow-up	0.38 (0.24 to 0.71)	0.35 (0.24 to 0.58)	0.42 (0.26 to 0.79)	0.35 (0.23 to 0.68)	0.36 (0.22 to 0.84)	0.44 (0.28 to 0.75)	0.45 (0.29 to 0.93)	0.33 (0.20 to 0.6)	0.33 (0.21 to 0.53)	0.38 (0.22 to 0.93)	0.31 (0.18 to 0.61)	0.31 (0.18 to 0.71)	0.34 (0.21 to 0.59)	0.36 (0.22 to 0.67)
	Change	0.07 (–0.10 to 0.30)	0.07 (–0.05 to 0.24)	0.09 (–0.10 to 0.36)	0.06 (–0.12 to 0.29)	0.06 (–0.12 to 0.3)	0.08 (–0.15 to 0.32)	0.06 (–0.18 to 0.37)	0.09 (–0.03 to 0.30)	0.09 (–0.03 to 0.24)	0.13 (–0.00 to 0.46)	0.08 (–0.02 to 0.31)	0.09 (–0.03 to 0.32)	0.07 (–0.03 to 0.26)	0.09 (–0.04 to 0.31)
CKD Prevalence^a^	Baseline	229 (5.4)	28 (2.4)	47 (5.9)	61 (6.3)	36 (7.7)	20 (6.0)	37 (6.7)	142 (4.4)	26 (2.5)	34 (5.9)	33 (5.9)	25 (7.8)	10 (3.3)	14 (3.1)
	Follow-up	360 (8.4)	53 (4.6)	78 (9.9)	119 (12.3)	49 (10.5)	18 (5.4)	43 (7.8)	291 (9.0)	55 (5.3)	84 (14.5)	59 (10.6)	50 (15.7)	14 (4.6)	29 (6.3)
CKD Incidence^a^^,^^b^		254 (5.9)	44 (3.8)	50 (6.3)	90 (9.3)	31 (6.6)	13 (3.9)	26 (4.7)	198 (6.0)	40 (3.9)	58 (10.0)	37 (6.6)	35 (11.0)	9 (3.0)	19 (4.1)
CKD Progression^a^^,^^c^		46 (1.1)	6 (0.5)	15 (1.9)	13 (1.3)	4 (0.9)	3 (0.9)	5 (0.9)	55 (1.7)	9 (0.9)	19 (3.3)	12 (2.1)	7 (2.2)	3 (1.0)	5 (1.1)

Abbreviations: KDIGO, Kidney Disease: Improving Global Outcomes; SA Surinamese, South-Asian Surinamese.

^a^ Measurements conducted in subgroup with available ACR samples.

^b^ Defined as eGFR <60 mL/min/1.73 m^2^ and/or ACR of < 3 mg/mmol.

^c^ Defined as either drop in CKD stage and decline of 25% or more in eGFR from the baseline estimates; as per KDIGO guideline.

Patterns of baseline and follow-up differences in secondary outcomes were mostly aligned with the overall differences in primary outcomes (Fig S2). CKD incidence also differs among ethnic groups. For example, more African Surinamese women developed CKD at the 6-year follow-up than men (9.0% vs 6.6%, respectively), whereas the opposite was observed in Ghanaian women and men (6.6% vs 11.0%, respectively). Nevertheless, more men in all groups experienced disease progression than did women.

### Regression Analyses

**Figure 2:**
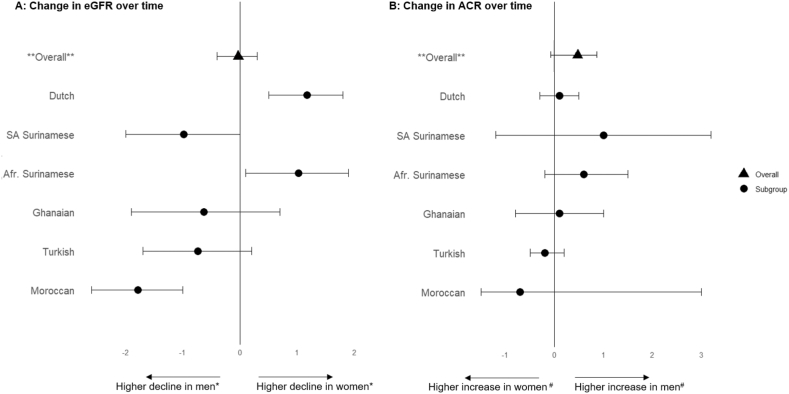
(A) Sex differences in change of estimated glomerular filtration rate GFR and (B) albumin to creatinine ratio over 6 years in men versus women in the total population and by ethnicity. ∗Higher decline of eGFR over time. ^#^Higher increase in ACR over time. Abbreviations: Afr. Surinamese, African Surinamese; SA Surinamese, South-Asian Surinamese.

Linear regression analyses showed no sex differences in the change in eGFR in the overall population after adjusting for main model variables (difference men vs women: –0.03 (–0.4 to 0.3); Table S6, Fig 2). However, the patterns differed across ethnic groups (*P* < 0.001). Dutch and African Surinamese men had a statistically significantly smaller decline in eGFR than women, suggesting a slower eGFR decline, whereas Moroccan and South-Asian Surinamese men had a significantly larger decline in eGFR over time compared with women. For ACR, the results indicated that change was larger in men than in women but was not associated with sex overall or across ethnic groups after adjustment. Our interpretation of sex differences in changes in eGFR and ACR did not change in the IPW analyses (Table S7). We also did not observe a clear pattern of sex differences when investigating the different age groups (Table S8).

In line with the larger estimated eGFR decline in South-Asian Surinamese men and African Surinamese women compared with their opposite sex counterparts, CKD incidence was also higher in these groups, but not overall (Table S9). Conversely, the estimated CKD progression was higher in men than in women overall but not across ethnic groups.

We observed little evidence for mediation of the associations observed for eGFR within the Dutch, South-Asian Surinamese, African Surinamese, and Moroccan groups (Tables 4 and S10). The proportion explained was low across all groups. The interpretation was similar when an alternative method was used (Table S11). Finally, the associations observed for CKD incidence and overall progression were not explained by mediators (Tables S12 and S13).

**Table 4:** Mediation Analysis of Sex Differences in Change in Estimated Glomerular Filtration Rate Over Time in the Total Population and by Ethnicity

	Overall	Dutch	SA Surinamese	Afr. Surinamese	Ghanaian	Turkish	Moroccan
Proportion of association explained [95% CI]							
Obesity	–0.14 (–4.70 to 4.85)	–0.01 (–0.5 to 0.02)	–0.14 (–1.25 to 0.26)	0.19 (–0.05 to 0.89)	0.21 (–4.21 to 3.37)	0.02 (–0.20 to 0.33)	0.01 (–0.08 to 0.11)
Alcohol Consumption	0.08 (–2.51 to 2.08)	–0.07 (–0.19 to 0.01)	–0.03 (–0.36 to 0.09)	0.05 (–0.01 to 0.30)	0.01 (–0.38 to 0.78)	0.01 (–0.29 to 0.29)	–0.004 (–0.05 to 0.03)
Physical Activity	–0.01 (–1.20 to 1.71)	–0.01 (–0.07 to 0.03)	–0.04 (–0.48 to 0.21)	–0.01 (–0.18 to 0.12)	–0.02 (–1.17 to 1.29)	0.007 (–0.38 to 0.51)	0.02 (–0.04 to 0.09)
Smoking	0.03 (–2.36 to 2.32)	–0.005 (–0.04 to 0.01)	–0.05 (–1.01 to 0.81)	0.02 (–0.35 to 0.34)	–0.06 (–2.40 to 1.73)	0.001 (–0.22 to 0.32)	–0.08 (–0.25 to 0.02)
Hypertension	0.28 (–8.34 to 7.85)	–0.13 (–0.32 to –0.02)	0.08 (–0.13 to 0.60)	0.003 (–0.14 to 0.14)	–0.004 (–0.56 to 0.47)	0.03 (–0.80 to 0.78)	0.05 (0.01 to 0.15)
Diabetes	0.03 (–1.07 to 1.62)	–0.02 (–0.09 to 0.02)	0.05 (–0.15 to 0.70)	0.01 (–0.08 to 0.15)	–0.001 (–0.67 to 0.56)	0.02 (–0.18 to 0.48)	–0.001 (–0.04 to 0.03)
CVD	0.01 (–0.60 to 0.67)	–0.02 (–0.08 to 0.01)	0.06 (–0.05 to 0.32)	0.04 (–0.05 to 0.2)	0.001 (–0.29 to 0.31)	0.01 (–0.27 to 0.30)	(.)

Abbreviations: 95% CI, 95% confidence interval; (.), numbers too small to depict.

## Discussion

### Main Findings

In the present study on sex differences in eGFR and ACR change in a multi-ethnic population, we observed no significant overall differences between men and women but found disparate results for some outcomes across different ethnic groups. Dutch and African Surinamese women had a larger eGFR decline than men, whereas Moroccan and South-Asian Surinamese men had a larger eGFR decline than women did. However, we did not find sex differences in ACR changes. Sex differences in secondary outcomes of CKD incidence were found in some ethnic groups in line with the sex differences in eGFR change, aside from both African and South-Asian Surinamese groups. Men had significantly higher CKD progression compared with women overall, but this was not the case across ethnic groups. There is limited evidence that traditional risk factors and health-related behaviors mediate the observed sex differences in all outcomes.

### Discussion of main findings

We found no differences in the changes in eGFR and ACR over time or in CKD incidence between women and men in the overall population. Our finding for eGFR aligns with other studies conducted in a healthy population in Israel and a general population in Japan that found no sex differences in the change in eGFR over time, although both studies observed a smaller annual decline in eGFR than the current study.^5^^,^^25^ Simultaneously, our findings contrast with the results of a Norwegian and Dutch study in healthy participants, which found that women had a slower decline in eGFR than men.^12^^,^^26^ Both of these studies, however, used varying outcome measures, such as measured GFR using iohexal clearance and estimated GFR using the Modification of Diet in Renal Disease Study equation. The lack of overall sex differences in ACR increase over time in our study is in contrast with further Dutch and Japanese studies that showed a higher increase in ACR in men compared with women.^15^^,^^27^ Possible explanations for this include differing study cohorts. For instance, Okada et al^15^ only included participants with CKD risk factors. Finally, our overall similar CKD incidence in women and men at 6 years follow-up did not align with a previous study conducted in Iran, which found higher incidences of CKD in women compared to men, possibly because of a longer follow-up time than our study.^28^ Nevertheless, our finding that disease progression in those with CKD at baseline occurred more frequently in men than in women is consistent with several studies regardless of follow-up time and outcome measurement.^10^^,^^11^^,^^15^^,^^29^

It is possible that the partial inconsistency in changes in eGFR and ACR between our study and others in the literature may, besides variations in kidney function measurement methods or longer follow-up times, be due to different countries and included subgroups.^6^ For instance, the HELIUS cohort used in this study was a younger cohort with preserved kidney function compared with other studies. The damaging effects of risk factors accumulate over time and may only become apparent in older age when compensatory mechanisms fail. Therefore, in younger populations, differences may be smaller. With longer follow-up periods, subtle differences may become more apparent. Importantly, the cohorts used in the current literature may not be as heterogeneous as this cohort. It is possible that differences in the direction of association across ethnic groups explain the overall lack of association between sex and change in eGFR.

Indeed, the patterns in eGFR across ethnic groups between women and men were inconsistent, ie, Dutch women had a larger decline in eGFR than men, whereas South-Asian women had a smaller decline than men. These associations between sex and change in eGFR were statistically significant in all ethnic groups, aside from the Ghanaian and Turkish groups. However, the results from the Dutch group did not align with a previously conducted PREVEND study in the Netherlands.^12^^,^^27^ Although Dutch men had higher eGFR at baseline, as in previous studies, they did not have a larger decline in eGFR than Dutch women. The opposing results may be explained by differences in the age of participants and the outcome measures used, as the PREVEND study estimated the eGFR slope using the MDRD study equation with more than 2 time points. Furthermore, differences in changes between ethnic groups align with a previous analysis showing large differences in CKD risk and risk of ESRD in minority versus majority groups.^30^ We hypothesized that differences in eGFR change between women and men across ethnic groups may be due to known differing cardiometabolic disease risk factors across groups.^19^^,^^31^, ^32^, ^33^ However, we found no evidence of the mediation of traditional CKD risk factors on the observed sex differences in eGFR decline or CKD incidence and progression across the groups in our study. Differences across ethnic groups may also relate to ethnicity-related variations in creatinine generation (eg, because of diet, body composition, and muscle mass).^34^ Alternatively, considering the small sample sizes in some ethnic groups, it is possible that the observed differences may be explained by chance variations. In either case, the results are hypothesis generating and suggest that potential differences and their underlying mechanisms should be explored further in future studies.

We also investigated whether health-related behaviors explained sex differences, as faster CKD progression in men has previously been attributed to smoking and higher body mass index as well as traditional CKD risk factors.^11^^,^^35^^,^^36^ However, in our study, we showed that health-related behaviors do not mediate the relationship between sex and change in eGFR within subgroups or overall CKD progression. In addition, another review argued that faster progression in men may be due to higher levels of albuminuria; however, we saw no evidence of this.^6^ The difference in findings may relate to the focus on more severe disease definitions; many studies have investigated progression to kidney replacement therapy or end-stage kidney disease, rather than a significant reduction in eGFR or ACR within a relatively healthy general population sample, as performed in our study.^11^ Hence, it is possible that findings may have differed if alternative outcome definitions had been used.

Future studies should further investigate the factors that explain the disparate sex differences within different ethnic groups. Several have previously been associated with prevalent CKD, such as diet and environment, or gender-related factors, such as stresses related to paid and unpaid work.^3^^,^^37^ Differing gendered roles or behaviors can determine the type of occupation, environmental exposure, and distribution of care (children, sick, or elderly), which in turn can affect an individual’s health outcomes.

Other aspects to consider include biological mechanisms, such as differences in (early) menopause between ethnic groups.^38^ For instance, sex differences in kidney function decline have been attributed to the effects of sex hormones.^39^^,^^40^ Masouri et al^41^ (2023) found that post-menopausal women had similar rates of CKD progression compared to age-matched men, showing that women no longer exposed to estrogen are at risk of faster kidney function decline. Further biological factors may also contribute to sex differences, including genetics, differences in kidney architecture, and inflammation. However, we were unable to include these in our general population study.

### Limitations

Our study includes limitations that should be considered when interpreting our results. First, our eGFR and ACR change estimates and secondary outcomes may have been subject to selection bias and attrition. Older people and women were selectively more likely to participate in HELIUS at baseline.^21^ Moreover, we found that those remaining in the study at 6 year follow-up had better CKD risk profile than those lost to follow-up; therefore, the magnitude of the changed in eGFR and ACR and associations observed may have been underestimated. However, applying weighting methods to our population subsets did not change our interpretation of the results. Nevertheless, this does not account for potential selections related to unobserved factors.^42^

Second, we used a single measurement of serum creatinine, urine albumin, and creatinine levels for our eGFR and ACR estimates, CKD, and other secondary outcomes, whereas it is recommended to use multiple samples to accurately diagnose the disease. Additionally, eGFR was calculated using serum creatinine levels, which is likely to be influenced by muscle mass and diet. Alternative markers, including cystatin C levels, are considered to be less influenced, but these are not available for all participants and all groups within our data. Although this limitation potentially affects the comparisons of baseline or follow-up values of ACR and eGFR across subgroups, this limitation may be less relevant for the interpretation of our main analyses that focus on changes within individuals.^43^ We have no evidence to indicate that major changes in diet or muscle mass occurred during the 6-year follow-up, or that this was more frequently observed in some groups than in others.^22^

Third, it is possible that some of the potential mediators in our analyses were imperfectly measured. For example, physical activity, smoking, and alcohol consumption were self-reported using a questionnaire. Self-reported data may suffer from certain biases, such as social desirability bias and measurement error. Thus, we may have underestimated the contribution of these factors to the sex differences observed within subgroups particularly in the mediation analyses.

Fourth, we acknowledge that findings on sex differences are limited to the groups that were included and may not be generalizable, eg, to undocumented people and people living in less urbanized areas. Moreover, classification of ethnicity based on municipality registered country of birth in combination with self-report, may not fully capture an individual’s lived experience and risk factor exposure.^23^ Applying alternative ethnic group categorization may result in differing findings.

Lastly, although we only have binary sex-related classifications in our cohort, we recognize that socio-cultural aspects (gender) between women and men may influence reported sex differences and that experiences of certain groups, such as those with differences in sex development, are not represented.

In conclusion, disparate patterns of change in eGFR were observed between women and men across ethnic groups. Dutch and African Surinamese women had a higher decline in eGFR than men, whereas South-Asian Surinamese and Moroccan men had a higher eGFR than women. Differences in sex disparities across ethnic groups illustrate the importance of considering ethnicity in the study of sex differences, the development of interventions, and the European context. The sex differences were not mediated by differences in traditional risk factors and health-related behaviors across any of the groups, implying that interventions focusing solely on these factors may not be sufficient. A more sex-specific, personalized approach may be more impactful; however, further research is needed to investigate the factors associated with the differences in kidney outcomes between men and women within population subgroups.
