# Supplementary material for: Sex Differences in 6-Year Change in eGFR and ACR in a Multiethnic Population: The HELIUS Study
Source: Kidney Med. 2026 Apr 30;8(7):101381. doi: 10.1016/j.xkme.2026.101381 (PMC13227188; doi:10.1016/j.xkme.2026.101381)
Supplement: Supplementary (PDF) — Figures S1-S3; Tables S1-S13 [file mmc1.pdf]

Supplementary Material

Figure S1: Simplified conceptual model

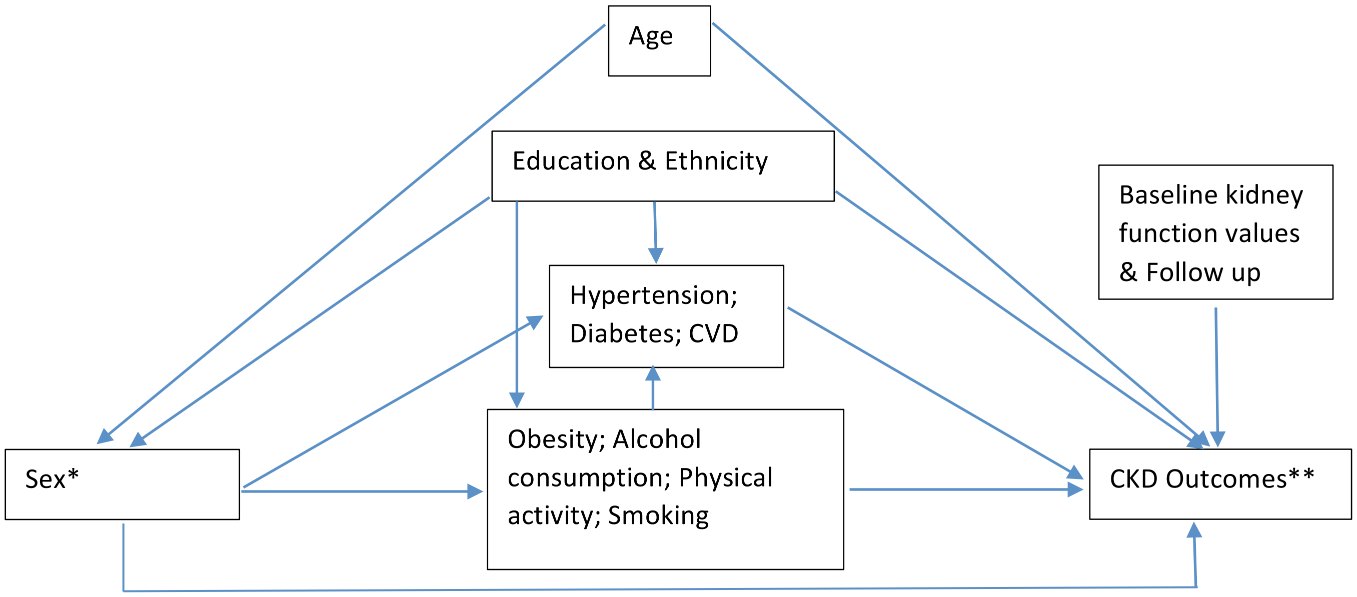

*Although we only have sex-related classifications of our cohort, we recognize that sociocultural aspects (gender) between women and men may influence sex differences. Therefore, we include a broader view of the concept of sex and consider gender-related aspects such as socially constructed roles, behaviours, power distributions, and identities.

**These include change in eGFR & ACR over time and CKD prevalence, incidence and progression.

CKD = chronic kidney disease, CVD = cardiovascular disease

Figure S2: Estimated glomerular filtration rate and albumin-to-creatinine ratio at baseline and 6-year follow up in women and men by ethnicity

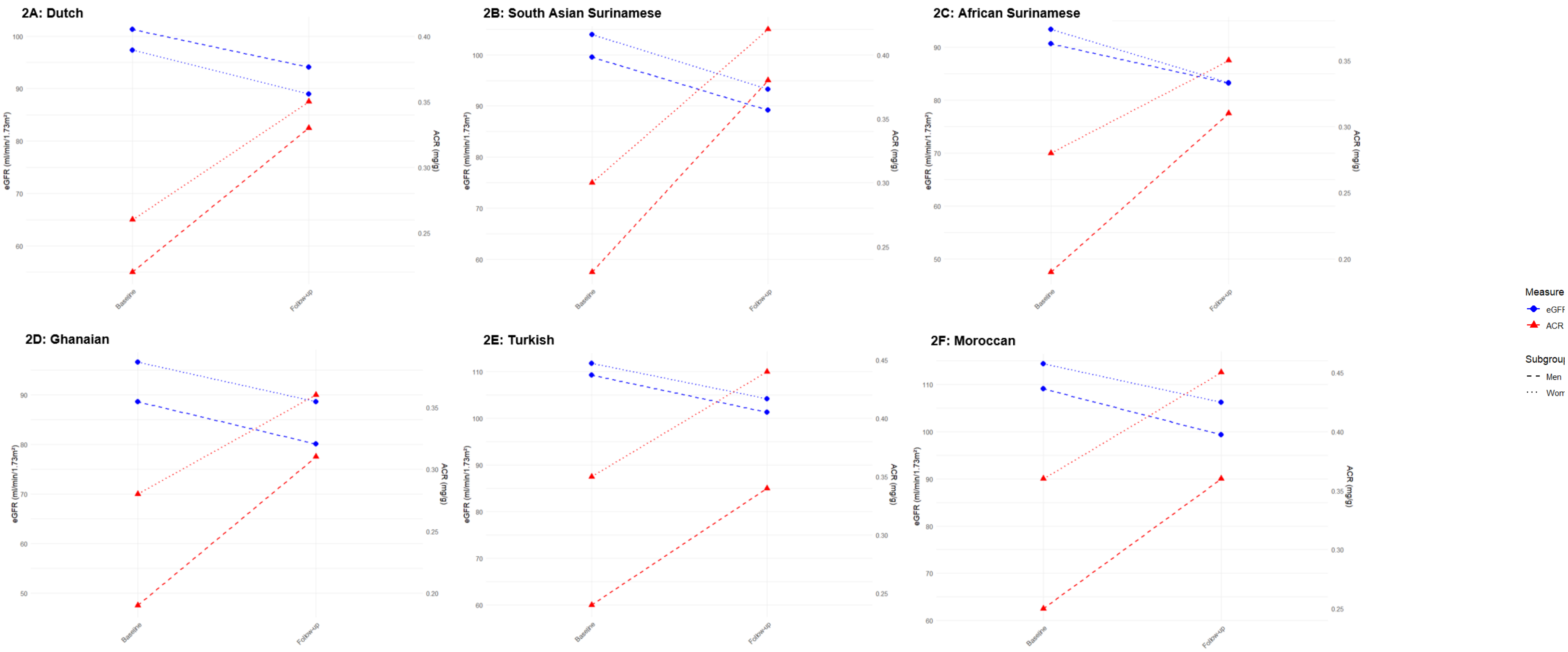

eGFR = estimated glomerular filtration rate, ACR = albumin to creatinine ratio

Figure S3: Chronic kidney disease prevalence at baseline and 6-year follow up in women and men in the total population and by ethnicity

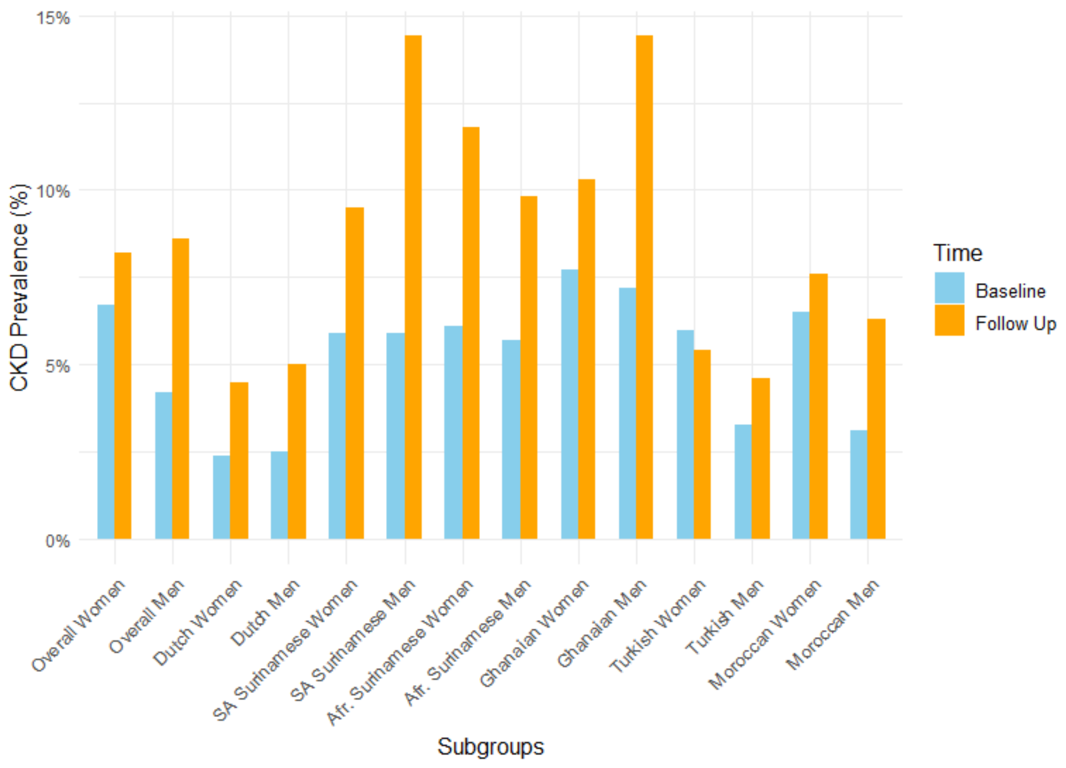

CKD = Chronic Kidney Disease, CKD Prevalence defined as eGFR <60 mL/min/1.73 m^2 and/or ACR of < 3 mg/mmol, SA Surinamese = South Asian Surinamese, Afr. Surinamese = African Surinamese

Table S1: Descriptive characteristics (ACR Subset)

		Women							Men						
		Overall (n=4283)	Dutch (n=1167)	SA Surinamese (n=790)	African Surinamese (n=966)	Ghanaian (n=469)	Turkish (n=336)	Moroccan (n=555)	Overall (n=3253)	Dutch (n=1031)	SA Surinamese (n=578)	African Surinamese (n=560)	Ghanaian (n=319)	Turkish (n=307)	Moroccan (n=458)
**Age (years)**		47.0 (11.3)	48.6 (11.7)	47.9 (11.3)	49.7 (11.2)	44.2 (9.3)	40.5 (10.8)	44.3 (10.4)	47.6 (11.5)	49.3 (11.5)	46.8 (12.4)	50.0 (11.1)	48.2 (10.3)	41.5 (11.3)	45.9 (10.1)
**Educational level**	Low	650 (15.2)	29 (2.5)	107 (13.6)	38 (3.9)	164 (35.6)	98 (29.8)	214 (38.6)	327 (10.1)	27 (2.6)	66 (11.4)	27 (4.9)	50 (15.9)	46 (15.1)	111 (24.5)
	Low-Medium	1027 (24.1)	144 (12.4)	280 (35.6)	270 (28.1)	175 (38.0)	63 (19.1)	95 (17.1)	839 (25.9)	122 (11.9)	169 (29.2)	217 (39.0)	138 (43.8)	90 (29.6)	103 (22.7)
	Medium high	1178 (27.7)	244 (21.0)	223 (28.4)	364 (37.9)	100 (21.7)	92 (28.0)	155 (28.0)	920 (28.5)	230 (22.4)	166 (28.7)	187 (33.6)	93 (29.5)	97 (31.9)	147 (32.4)
	High	1398 (32.9)	745 (64.1)	176 (22.4)	289 (30.1)	22 (4.8)	76 (23.1)	90 (16.2)	1145 (35.4)	646 (63.0)	177 (30.6)	125 (22.5)	34 (10.8)	71 (23.3)	92 (20.3)
**Alcohol consumption**	Low	3156 (74.2)	393 (33.8)	685 (87.4)	813 (84.9)	412 (89.2)	311 (92.6)	542 (98.0)	2220 (68.6)	382 (37.1)	457 (79.3)	410 (73.9)	272 (86.3)	273 (89.5)	426 (93.6)
	Moderate	733 (17.2)	484 (41.6)	77 (9.8)	106 (11.1)	40 (8.6)	19 (5.7)	7 (1.3)	795 (24.6)	518 (50.3)	82 (14.2)	115 (20.7)	42 (13.3)	20 (6.5)	18 (4.0)
	High	366 (8.6)	285 (24.5)	22 (2.8)	39 (4.1)	10 (2.2)	6 (1.8)	4 (0.7)	220 (6.8)	129 (12.5)	37 (6.4)	30 (5.4)	1 (0.3)	12 (3.9)	11 (2.4)
**Physical activity***	Yes	2490 (58.2)	914 (78.4)	396 (50.3)	568 (58.8)	234 (50.0)	129 (38.5)	249 (44.9)	2117 (65.2)	746 (72.4)	341 (59.1)	396 (70.08)	110 (34.5)	155 (50.5)	270 (59.1)
	No	1788 (41.8)	252 (21.6)	392 (49.7)	398 (41.2)	234 (50.0)	206 (61.5)	306 (55.1)	1132 (34.8)	284 (27.6)	236 (40.9)	163 (29.2)	209 (65.5)	152 (49.5)	187 (40.9)
**Obesity**	Yes	1111 (25.9)	101 (8.7)	176 (22.3)	331 (34.3)	202 (43.2)	94 (28.0)	207 (37.3)	436 (13.4)	102 (9.9)	59 (10.2)	70 (12.5)	51 (16.0)	77 (25.1)	77 (16.8)
	No	3168 (74.1)	1065 (91.3)	614 (77.7)	633 (65.7)	266 (56.8)	242 (72.0)	348 (62.7)	2815 (86.6)	929 (90.1)	518 (89.8)	490 (87.5)	267 (84.0)	230 (74.9)	381 (83.2)
**Smoking**	Current	694	237	135 (17.2)	196 (20.4)	9 (1.9)	95	22 (4.0)	853	229	184 (31.9)	222 (39.9)	30 (9.5)	101	87 (19.0)

		(16.3)	(20.4)				(28.5)		(26.3)	(22.2)				(33.1)	
	Former/Never	3571 (83.7)	927 (79.6)	651 (82.8)	766 (79.6)	458 (98.1)	238 (71.5)	531 (96.0)	2389 (73.7)	801 (77.8)	393 (68.1)	334 (60.1)	287 (90.5)	204 (66.9)	370 (81.0)
**Hypertension**	Yes	1287 (30.1)	200 (17.1)	263 (33.3)	440 (45.5)	226 (48.2)	53 (15.8)	105 (18.9)	1211 (37.2)	339 (32.9)	249 (43.1)	249 (44.5)	182 (57.1)	79 (25.7)	113 (24.7)
	No	2996 (69.9)	967 (82.9)	527 (66.7)	526 (54.5)	243 (48.2)	283 (84.2)	450 (81.1)	2042 (62.7)	692 (67.1)	329 (56.9)	311 (55.5)	137 (42.9)	228 (74.3)	345 (75.3)
**Diabetes**	Yes	366 (8.5)	21 (1.8)	125 (15.8)	89 (9.2)	46 (9.8)	19 (5.7)	66 (11.9)	340 (10.5)	47 (4.6)	129 (22.3)	42 (7.5)	37 (11.6)	22 (7.2)	63 (13.8)
	No	3917 (91.5)	1146 (98.2)	665 (84.2)	877 (90.8)	423 (90.2)	317 (94.3)	489 (88.1)	2913 (89.5)	984 (95.4)	449 (77.7)	518 (92.5)	282 (88.4)	285 (92.8)	395 (86.2)
**CVD**	Yes	557 (13.0)	63 (5.4)	131 (16.6)	152 (15.7)	70 (14.9)	58 (17.3)	83 (15.0)	466 (14.3)	84 (8.1)	131 (22.7)	80 (14.3)	42 (13.2)	51 (16.6)	78 (17.0)
	No	3726 (87.0)	1104 (94.6)	659 (83.4)	814 (84.3)	399 (85.1)	278 (82.7)	472 (85.0)	2787 (85.7)	947 (91.9)	447 (77.3)	480 (85.7)	277 (86.8)	256 (83.4)	380 (83.0)

CVD = Cardiovascular disease; SA Surinamese = South Asian Surinamese; Afr. Surinamese = African Surinamese, * Defined by adherence to Dutch physical activity guidelines.

Table S2: Descriptive characteristics at follow up (eGFR Subset)

		**Women**							**Men**						
		Overall (n=5713)	Dutch (n=1548)	SA Surinamese (n=962)	African Surinamese (n=1315)	Ghanaian (n=478)	Turkish (n=547)	Moroccan (n=863)	Overall (n=4407)	Dutch (n=1401)	SA Surinamese (n=734)	African Surinamese (n=759)	Ghanaian (n=324)	Turkish (n=498)	Moroccan (n=691)
**Age (years)**		52.5 (12.4)	53.9 (13.2)	53.9 (12.2)	55.7 (11.5)	50.8 (9.8)	47.7 (11.4)	47.7 (12.0)	52.8 (12.6)	54.3 (13.1)	52.6 (13.0)	56.0 (11.6)	54.9 (10.5)	47.6 (11.4)	49.4 (11.9)
**Follow up duration**		83.7 (17.5)	84.4 (19.6)	91.2 (18.4)	84.8 (17.6)	80.3 (11.1)	77.5 (13.0)	78.5 (14.3)	83.1 (17.6)	83.1 (20.2)	90.3 (18.1)	83.7 (17.1)	80.3 (10.4)	78.4 (13.3)	79.6 (15.1)
**Alcohol consumption**	Low	4070 (80.5)	568 (45.8)	769 (90.5)	947 (86.8)	421 (89.6)	519 (94.9)	846 (98.4)	2972 (77.2)	558 (50.3)	532 (84.0)	500 (80.4)	264 (86.3)	455 (91.4)	663 (97.1)
	Moderate	716 (14.2)	471 (38.0)	62 (7.3)	115 (10.5)	39 (8.3)	20 (3.7)	9 (1.0)	743 (19.3)	465 (41.9)	87 (13.7)	102 (16.4)	39 (12.7)	35 (7.0)	15 (2.2)

	High	273 (5.4)	202 (16.3)	19 (2.2)	29 (2.7)	10 (2.1)	8 (1.5)	5 (0.6)	136 (3.5)	86 (7.8)	14 (2.2)	20 (3.2)	3 (1.0)	8 (1.6)	5 (0.7)
**Obesity**	Yes	1801 (31.5)	183 (11.8)	241 (25.1)	540 (41.1)	269 (56.3)	221 (40.4)	347 (40.2)	781 (17.7)	154 (11.0)	116 (15.8)	133 (17.5)	67 (20.7)	156 (31.5)	155 (22.4)
	No	3911 (68.5)	1365 (88.2)	720 (74.9)	775 (58.9)	209 (43.7)	326 (59.6)	516 (59.8)	3624 (82.8)	1247 (89.0)	618 (84.2)	626 (82.5)	257 (79.3)	340 (68.5)	536 (77.6)
**Smoking**	Current	788 (13.8)	219 (14.1)	129 (13.4)	248 (18.9)	14 (2.9)	140 (25.6)	38 (4.4)	1068 (24.2)	249 (17.8)	216 (29.4)	277 (36.5)	31 (9.6)	178 (35.7)	117 (16.9)
	Former/Never	4925 (86.2)	1329 (85.9)	833 (86.6)	1067 (81.1)	464 (97.1)	407 (74.4)	825 (95.6)	3339 (75.8)	1152 (82.2)	518 (70.6)	482 (63.5)	293 (90.4)	320 (64.3)	574 (83.1)
**Hypertension**	Yes	2023 (35.4)	357 (23.1)	387 (40.2)	679 (51.6)	274 (57.3)	136 (24.9)	190 (22.0)	1786 (40.5)	495 (35.3)	338 (46.0)	379 (49.9)	204 (63.0)	156 (31.3)	214 (31.0)
	No	3690 (64.6)	1191 (76.9)	575 (59.8)	636 (48.4)	204 (42.7)	411 (75.1)	673 (78.0)	2621 (59.5)	906 (64.7)	396 (54.0)	380 (50.1)	120 (37.0)	342 (68.7)	477 (69.0)
**Diabetes**	Yes	707 (12.8)	52 (3.4)	213 (22.1)	192 (14.6)	78 (16.3)	54 (9.8)	118 (13.7)	655 (14.9)	95 (6.8)	202 (27.5)	111 (14.6)	57 (17.8)	76 (15.3)	114 (16.5)
	No	5006 (87.6)	1496 (96.6)	749 (77.9)	1123 (85.4)	400 (83.7)	493 (90.1)	745 (86.3)	3752 (85.1)	1306 (93.2)	532 (72.5)	648 (85.4)	267 (82.4)	422 (84.7)	577 (83.5)
**CVD**	Yes	317 (5.5)	65 (4.2)	85 (8.8)	78 (5.9)	21 (4.4)	33 (6.0)	35 (4.0)	432 (9.8)	118 (8.4)	121 (16.5)	76 (10.0)	24 (7.4)	45 (9.0)	48 (6.9)
	No	5396 (94.5)	1483 (95.8)	877 (91.2)	1237 (94.1)	547 (95.6)	514 (94.0)	828 (96.0)	3975 (90.2)	1283 (91.6)	613 (83.5)	683 (90.0)	300 (92.6)	453 (91.0)	643 (93.1)

CVD = Cardiovascular disease; eGFR = estimated glomerular filtration rate; SA Surinamese = South Asian Surinamese; Afr. Surinamese = African Surinamese , eGFR subset = measurements conducted in subset with eGFR measurements

Table S3: Descriptive characteristics at follow up (ACR Subset)

		Women							Men						
		Overall (n=4283)	Dutch (n=1167)	SA Surinamese (n=790)	African Surinamese (n=966)	Ghanaian (n=469)	Turkish (n=336)	Moroccan (n=555)	Overall (n=3253)	Dutch (n=1031)	SA Surinamese (n=578)	African Surinamese (n=560)	Ghanaian (n=319)	Turkish (n=307)	Moroccan (n=458)
**Age**		53.5 (11.4)	55.1 (11.6)	54.7 (11.3)	56.1 (11.3)	50.9 (9.5)	46.8 (10.8)	50.5 (10.3)	54.1 (11.5)	55.8 (11.5)	53.5 (12.4)	56.4 (11.2)	54.9 (10.4)	47.8 (11.1)	52.1 (10.0)
**Follow up duration**		87.6 (17.0)	88.2 (19.1)	95.3 (16.8)	88.0 (17.5)	80.1 (11.1)	81.8 (12.9)	84.7 (12.7)	87.4 (16.9)	88.1 (19.6)	94.4 (16.8)	86.8 (16.6)	80.3 (10.4)	83.1 (13.0)	86.0 (13.4)
**Alcohol consumption**	Low	3076 (79.3)	456 (45.5)	637 (90.9)	711 (86.1)	413 (89.6)	315 (93.8)	544 (98.0)	2204 (76.0)	434 (50.0)	422 (83.9)	376 (80.3)	259 (86.0)	278 (90.6)	435 (96.0)
	Moderate	574 (14.8)	379 (37.8)	46 (6.6)	89 (10.8)	38 (8.2)	15 (4.5)	7 (1.2)	595 (20.5)	373 (42.8)	71 (14.1)	75 (16.0)	39 (13.0)	23 (7.5)	14 (3.1)
	High	231 (5.9)	167 (16.7)	18 (2.6)	26 (3.1)	10 (2.2)	6 (1.8)	4 (0.7)	101 (3.4)	61 (7.0)	10 (2.0)	17 (3.6)	3 (1.0)	6 (2.0)	4 (0.9)
**Obesity**	Yes	1360 (31.8)	147 (12.6)	195 (24.7)	388 (40.2)	264 (56.3)	124 (36.9)	242 (43.6)	566 (17.4)	119 (11.5)	89 (15.4)	87 (15.5)	66 (20.7)	101 (33.0)	104 (22.7)
	No	2922 (68.2)	1020 (87.4)	594 (75.3)	578 (59.8)	205 (43.7)	212 (63.1)	313 (56.4)	2686 (82.6)	912 (88.5)	489 (84.6)	473 (84.5)	253 (79.3)	205 (67.0)	354 (77.3)
**Smoking**	Current	565 (13.2)	162 (13.9)	98 (12.4)	177 (18.3)	14 (3.0)	92 (27.4)	22 (4.0)	756 (23.2)	169 (16.4)	164 (28.4)	201 (35.9)	31 (9.7)	114 (37.1)	77 (16.8)
	Former/Never	3718 (86.8)	1005 (86.1)	692 (87.6)	789 (81.7)	455 (97.0)	244 (72.6)	533 (96.0)	2497 (76.8)	862 (83.6)	414 (71.6)	359 (64.1)	288 (90.3)	193 (62.9)	381 (83.2)
**Hypertension**	Yes	1578 (36.8)	275 (23.6)	323 (40.9)	495 (51.2)	270 (57.6)	73 (21.7)	142 (25.6)	1402 (43.1)	378 (36.7)	287 (49.7)	281 (50.2)	202 (63.3)	99 (32.2)	155 933.8)
	No	2705 (63.2)	892 (76.4)	467 (59.1)	471 (48.8)	199 (42.4)	263 (78.3)	413 (74.4)	1851 (56.9)	653 (63.3)	291 (50.3)	279 (49.8)	117 (36.7)	208 (67.8)	303 (66.2)
**Diabetes**	Yes	537 (12.5)	38 (3.3)	178 (22.5)	178 (22.5)	78 (16.6)	27 (8.0)	89 (16.0)	482 (14.8)	69 (6.7)	160 (27.7)	69 (12.3)	56 (17.6)	43 (14.0)	85 (18.6)
	No	3746	1129	612 (77.5)	612 (77.5)	391	309	466	2771	962	418 (72.3)	491 (87.7)	263	264	373

		(87.5)	(96.7)			(83.4)	(92.0)	(84.0)	(85.2)	(93.3)			(82.4)	(86.0)	(81.4)
**CVD**	Yes	252 (5.8)	48 (4.1)	75 (9.5)	64 (6.6)	21 (4.5)	18 (5.4)	26 (4.7)	341 (10.5)	89 (8.6)	109 (18.9)	57 (10.2)	22 (6.9)	29 (9.4)	35 (7.6)
	No	4031 (94.1)	1119 (95.9)	715 (90.5)	902 (93.4)	448 (95.5)	318 (94.6)	529 (95.3)	2912 (89.5)	942 (91.4)	469 (81.1)	503 (89.8)	297 (93.1)	278 (90.6)	423 (92.4)

ACR subset = Measurements conducted in subgroup with available ACR samples *, CVD = Cardiovascular disease; ACR = Albumin to creatine ratio, SA Surinamese = South Asian Surinamese; Afr. Surinamese = African Surinamese

Table S4: Baseline characteristics of lost to follow up population

		**Women**							**Men**						
		Overall (n=6454)	Dutch (n=895)	SA Surinamese (n=676)	African Surinamese (n=1140)	Ghanaian (n=889)	Turkish (n=1385)	Moroccan (n=1469)	Overall (n=4528)	Dutch (n=657)	SA Surinamese (n=613)	African Surinamese (n=825)	Ghanaian (n=543)	Turkish (n=1097)	Moroccan (n=793)
**Age (years)**		41.6 (13.5)	42.2 (13.5)	44.4 (14.5)	45.9 (13.0)	42.8 (11.3)	39.2(12.4)	38.1 (13.2)	43.4 (13.7)	44.6 (15.2)	43.5 (14.4)	46.6 (13.8)	46.2 (12.0)	40.5 (12.4)	40.8 (13.3)
**Educational level**	Low	1575 (24.6)	40 (4.5)	118 (17.6)	65 (5.7)	325 (37.2)	529 (38.6)	498 (34.1)	803 (17.9)	33 (5.0)	91 (14.9)	69 (8.4)	84 (15.7)	306 (28.1)	220 (28.2)
	Low-Medium	1605 (25.1)	161 (18.2)	221 (32.9)	408 (36.1)	314 (36.0)	288 (21.0)	213 (14.6)	1428 (31.8)	113 (17.2)	217 (35.6)	342 (41.9)	248 (46.4)	337 (30.9)	171 (21.9)
	Medium high	1860 (29.1)	182 (20.5)	176 (26.2)	406 (35.9)	193 (22.1)	390 (28.4)	513 (35.1)	1368 (30.5)	165 (25.2)	196 (32.1)	284 (34.8)	160 (29.9)	303 (27.8)	260 (33.3)
	High	1354 (21.2)	504 (56.8)	157 (23.4)	252 (22.2)	41 (4.7)	164 (12.0)	236 (16.2)	889 (19.8)	345 (52.6)	106 (17.4)	122 (14.9)	43 (8.0)	144 (13.2)	129 (16.5)
**Alcohol consumption**	Low	5407 (84.3)	333 (37.3)	574 (85.8)	928 (82.1)	799 (90.5)	1334 (96.9)	1439 (98.4)	3556 (79.2)	273 (41.7)	475 (77.9)	628 (77.1)	472 (87.9)	971 (89.7)	737 (93.5)
	Moderate	676 (10.5)	336 (37.7)	70 (10.5)	150 (13.3)	71 (8.0)	32 (2.3)	17 (1.2)	664 (14.8)	283 (43.2)	79 (12.9)	145 (17.8)	63 (11.7)	64 (5.9)	30 (3.8)
	High	331 (5.2)	223 (25.0)	25 (3.7)	53 (4.7)	13 (1.5)	11 (0.8)	6 (0.4)	268 (6.0)	99 (15.1)	56 (9.2)	42 (5.2)	2 (0.4)	48 (4.4)	21 (2.7)
**Physical activity***	Yes	3088 (47.9)	677 (75.7)	334 (49.5)	621 (54.5)	413 (46.5)	466 (33.7)	577 (39.4)	2720 (60.2)	479 (73.0)	342 (56.0)	570 (69.2)	338 (62.2)	547 (50.0)	444 (56.1)
	No	3359 (52.1)	217 (24.3)	341 (50.5)	518 (45.5)	476 (53.5)	918 (66.3)	889 (60.6)	1798 (39.8)	177 (27.0)	269 (44.0)	254 (30.8)	205 (37.7)	546 (49.9)	347 (43.9)

**Obesity**	Yes	2278 (35.3)	115 (12.9)	173 (25.6)	452 (39.7)	406 (45.7)	607 (43.9)	525 (35.8)	925 (20.4)	77 (11.7)	102 (16.6)	165 (20.0)	99 (18.2)	323 (29.5)	159 (20.1)
	No	4167 (64.6)	779 (87.1)	502 (74.4)	685 (60.2)	482 (54.3)	776 (56.1)	943 (64.2)	3599 (79.5)	579 (88.3)	511 (83.4)	659 (80.0)	444 (81.8)	773 (70.5)	633 (79.9)
**Smoking**	Current	1254 (19.5)	251 (28.2)	148 (21.9)	326 (28.7)	24 (2.7)	417 (30.3)	88 (6.0)	1676 (37.2)	214 (32.6)	298 (48.8)	374 (45.6)	34 (6.3)	492 (45.2)	264 (33.5)
	Former/Never	5178 (80.5)	640 (71.8)	527 (78.1)	811 (71.3)	861 (97.3)	961 (69.7)	1378 (94.0)	2831 (62.8)	442 (67.4)	313 (51.2)	446 (54.4)	508 (93.7)	597 (54.8)	525 (66.5)
**Hypertension**	Yes	2244 (34.8)	215 (24.0)	295 (43.6)	574 (50.4)	476 (53.5)	383 (27.7)	301 (20.5)	1863 (41.2)	249 (37.9)	277 (45.2)	409 (49.6)	340 (62.6)	363 (33.1)	225 (28.4)
	No	4207 (65.2)	680 (76.0)	381 (56.4)	566 (49.6)	413 (46.5)	1000 (27.7)	1167 (79.5)	2664 (58.8)	408 (62.1)	336 (54.8)	416 (50.4)	203 (37.4)	733 (66.9)	568 (71.6)
**Diabetes**	Yes	725 (11.2)	25 (2.8)	144 (21.3)	162 (14.2)	88 (9.9)	142 (10.3)	164 (11.2)	617 (13.6)	43 (6.5)	135 (22.0)	112 (13.6)	92 (16.9)	134 (12.2)	101 (12.7)
	No	5729 (88.8)	870 (97.2)	532 (78.7)	978 (85.8)	801 (90.1)	1243 (89.7)	1305 (88.8)	3911 (86.4)	614 (93.5)	478 (78.0)	713 (86.4)	451 (83.1)	963 (87.8)	692 (87.3)
**CVD**	Yes	1089 (16.9)	78 (8.7)	151 (22.3)	203 (17.8)	116 (13.0)	317 (22.9)	224 (15.2)	753 (16.6)	78 (11.9)	154 (25.1)	114 (13.8)	65 (12.0)	203 (18.5)	139 (17.5)
	No	5265 (83.1)	817 (91.3)	525 (77.7)	937 (82.2)	773 (87.0)	1068 (77.1)	1245 (84.7)	3775 (83.4)	579 (88.1)	459 (74.9)	711 (86.2)	478 (88.0)	894 (81.5)	654 (82.5)

CVD = Cardiovascular disease; SA Surinamese = South Asian Surinamese; * Defined by adherence to Dutch physical activity guidelines.

Table S5: Kidney function outcomes with Inverse probability weighting method (IPW)

		Women							Men						
		Overall	Dutch	SA Surinamese	Afr. Surinamese	Ghanaian	Turkish	Moroccan	Overall	Dutch	SA Surinamese	Afr. Surinamese	Ghanaian	Turkish	Moroccan
**eGFR [mL/min/1.73 m^2]**	Baseline	104.36 (91.26-114.57)	98.05 (85.73-107.19)	103.32 (92.72-111.96)	94.38 (82.23-105.14)	97.50 (84.76-108.10)	112.42 (105.09-12.68)	115.30 (106.99-123.59)	101.72 (89.54-111.48)	100.91 (90.67-110.46)	100.01 (88.13-109.58)	91.48 (80.13-102.54)	89.53 (79.06-100.40)	108.85 (100.64-117.29)	109.05 (101.21-117.40)
	Follow up	91.57 (79.90-103.61)	87.94 (78.18-98.51)	92.19 (82.04-102.12)	82.53 (73.40-95.00)	87.77 (77.81-98.56)	103.07 (94.99-110.88)	105.05 (96.80-114.34)	91.57 (79.75-102.65)	93.05 (82.49-102.81)	88.50 (77.61-100.36)	82.24 (72.33-94.31)	79.09 (70.13-92-80	100.26 (90.42-108.32)	98.15 (89.80-107.19)
	Change	-8.11 (-13/92--3.95)	-7.80 (-13.58-2.71)	-9.05 (-15.59- -4.75)	-9.03 (-14.82- -3.65)	-7.48 (-13.17- -2.99)	-7.83 (-12.54- -4.78)	-7.73 (-12.06- -5.11)	-7.86 (-13.97- -3.79)	-6.93 (-12.24--3.05)	-9.86 (-16.67- -4.44)	-6.84 (-13.51- -1.95)	-7.03 (-13.14- -1.15)	-8.29 (-14.14- -4.88)	-9.04 (-14.32- -5.8)
**ACR [mg/mmol]** #	Baseline	0.31 (0.19-0.61)	0.27 (0.17-0.42)	0.31 (0.19-0.62)	0.29 (0.18-0.57)	0.28 (0.18-0.62)	0.37 (0.22-0.74)	0.39 (0.23-0.81)	0.23 (0.14-0.44)	0.22 (0.14-0.37)	0.25 (0.14-0.55)	0.22 (0.13-0.45)	0.19 (0.11-0.40)	0.25 (0.15-0.46)	0.26 (0.16-0.49)
	Follow up	0.38 (0.25-0.71)	0.35 (0.24-0.59)	0.41 (0.25-0.79)	0.35 (0.22-0.67)	0.36 (0.22-0.84)	0.43 (0.27-0.72)	0.53 (0.32-0.85)	0.33 (0.20-0.62)	0.32 (0.20-0.52)	0.38 (0.22-0.96)	0.31 (0.17-0.61)	0.31 (0.18-0.70)	0.36 (0.22-0.64)	0.41 (0.24-0.63)
	Change	0.07 (-0.10-0.30)	0.07 (-0.05-0.24)	0.09 (-0.10-0.34)	0.06 (-0.12-0.28)	0.06 (-0.12-0.3)	0.07 (-0.18-0.33)	0.10 (-0.12-0.39)	0.09 (-0.03-0.29)	0.09 (-0.03-0.24)	0.14 (-0.01-0.47)	0.08 (-0.02-0.31)	0.09 (-0.03-0.31)	0.08 (-0.03-0.28)	0.10 (-0.02-0.32)
**CKD Prevalence**#	Baseline	6.3	2.9	7.3	6.7	9.0	7.1	6.6	5.6	3.2	8.6	6.7	8.3	5.1	4.1
	Follow up	9.3	5.6	10.8	14.2	10.6	6.9	8.0	10.0	6.0	15.8	14.2	16.0	5.6	4.1
**CKD Incidence*,**#		5.8	4.6	5.9	10.0	5.6	3.2	4.3	5.8	3.9	10.0	7.4	9.5	3.4	2.7
**CKD Progression**,**#		0.5	0.4	0.1	0.6	0.9	0.3	0.3	0.8	0.4	1.8	1.2	0.2	0.2	0.3

eGFR = estimated glomerular filtration rate, ACR = Albumin to creatinine ratio, SA Surinamese = South Asian Surinamese; Afr. Surinamese = African Surinamese, CKD = chronic kidney disease, #Measurements conducted in subgroup with available ACR samples *Defined as eGFR <60 mL/min/1.73 m^2 and/or ACR of < 3 mg/mmol. **Defined as either drop in CKD stage and decline of 25% or more in eGFR from the baseline estimates; as per KDIGO guideline.

Table S6: Sex differences in change of estimated glomerular filtration rate GFR and albumin to creatinine ratio over 6 years in men versus women in the total population and by ethnicity

	**Overall**		**Dutch**		**SA Surinamese**		**Afr. Surinamese**		**Ghanaian**		**Turkish**		**Moroccan**		**P value interaction term**
	β [95% CI]	p-value	β [95% CI]	p-value	β [95% CI]	p-value	β [95% CI]	p-value	β [95% CI]	p-value	β [95% CI]	p-value	β [95% CI]	p-value	
**Change in eGFR**															
**Unadjusted**	0.13 (-0.2-0.5)	0.477	0.05 (-0.6-0.7)	0.870	0.12 (-0.9-1.1)	0.834	1.43 (0.5-2.3)	0.001	0.57 (-0.7-1.9)	0.387	-0.46 (-1.4-0.5)	0.339	-1.34 (-2.1- -0.6)	0.001	0.005
**Model 1**	0.01 (-0.4-0.4)	0.966	0.70 (0.1-1.3)	0.031	-0.55 (-1.6-0.4)	0.280	1.06 (0.2-1.9)	0.015	-0.88 (-2.1-0.4)	0.162	-0.81 (-1.7-0.1)	0.092	-1.69 (-2.5- -0.9)	0.000	0.000
**Model 2**	-0.03 (-0.4-0.3)	0.850	1.17 (0.5-1.8)	0.000	-0.99 (-2.0-0.0)	0.053	1.02 (0.1-1.9)	0.019	-0.63 (-1.9-0.7)	0.347	-0.74 (-1.7-0.2)	0.123	-1.79 (-2.6- -1.0)	0.000	0.000
**Change in ACR***															
**Unadjusted**	0.49 (0.3-0.9)	0.02	0.04 (-0.4-0.4)	0.846	0.9 (-1.3-3.1)	0.424	0.7 (-0.2-1.5)	0.127	0.4 (-0.6-1.3)	0.465	0.9 (-0.5-2.3)	0.197	2.3 (-0.4-5.1)	0.098	0.007
**Model 1**	0.5 (-0.1-1.0)	0.07	0.1(-0.3-0.5)	0.652	0.9 (-1.2-3.1)	0.413	0.6 (-0.2-1.5)	0.126	-0.1 (-0.9-0.7)	0.802	-0.2 (-0.6-0.2)	0.351	1.1 (-1.1-3.3)	0.320	0.699
**Model 2**	0.6 (-0.1-1.1)	0.09	0.1 (-0.3-0.5)	0.711	1.0 (-1.2-3.2)	0.364	0.6 (-0.2-1.5)	0.141	0.1 (-0.8-1.0)	0.813	-0.2 (-0.5-0.2)	0.391	0.7 (-1.5-3.0)	0.519	0.699

*Analyses conducted in subgroup with available ACR measurements at follow up n=7536; Model 1: Adjusted for baseline kidney function values & follow up duration; Model 2: Model 1 + age, education & ethnicity; eGFR = estimated glomerular filtration rate; ACR = Albumin to creatinine ratio, SA Surinamese = South Asian Surinamese; Afr. Surinamese = African Surinamese

Table S7: Main model analysis with Inverse probability weighting method (IPW)

	Overall		Dutch		SA Surinamese		Afr. Surinamese		Ghanaian		Turkish		Moroccan		P value interaction term
	β [95% CI]	p-value	β [95% CI]	p-value	β [95% CI]	p-value	β [95% CI]	p-value	β [95% CI]	p-value	β [95% CI]	p-value	β [95% CI]	p-value	p-value
**Change in eGFR**															
**Model 2**	-0.03 (-0.38-0.33)	0.894	1.16 (0.5-1.8)	0.000	-1.0 (-2.0- -0.01)	0.048	1.09 (0.2-1.9)	0.013	-0.55 (-1.84-0.74)	0.401	-0.71 (-1.7-0.2)	0.137	-1.8 (-2.6- -1.0)	0.000	0.000
**Change in ACR***															
**Model 2**	0.50 (-0.07-1.06)	0.085	0.1 (-0.3-0.5)	0.716	1.08 (-1.1-3.3)	0.330	0.6 (-0.2-1.5)	0.126	0.16 (-0.87-1.19)	0.754	-0.18 (-0.58-0.21)	0.352	0.7 (-1.5-2.9)	0.524	0.699

eGFR = estimated glomerular filtration rate, ACR = Albumin to creatinine ratio, SA Surinamese = South Asian Surinamese; Afr. Surinamese = African Surinamese; *Measurements conducted in subgroup with available ACR samples

Table S8: Sensitivity Analyses stratified by age - main model analyses

**Model 2**	Overall		Dutch		SA Surinamese		Afr. Surinamese		Ghanaian		Turkish		Moroccan	
	β (95% CI)	p-value	β (95% CI)	p-value	β (95% CI)	p-value	β (95% CI)	p-value	β (95% CI)	p-value	β (95% CI)	p-value	β (95% CI)	p-value
**Change in eGFR**														
**≤50 years**	-0.07 (-0.53-0.38)	0.757	1.55 (0.66-2.44)	0.001	-0.07 (-1.30-1.16)	0.911	0.50 (-0.79-1.78)	0.448	-0.16 (-1.84-1.52)	0.851	-0.706 (-1.81-0.40)	0.210	-1.66 (-2.57--0.76)	0.000
**>50 years**	0.07 (-0.50-0.63)	0.812	0.37 (-0.51-1.26)	0.407	-1.70 (-3.40- -0.01)	0.049	1.49 (0.34-2.64)	0.011	-1.36 (-3.46-0.75)	0.206	-0.70 (2.41-1.02)	0.426	-2.11 (-3.73--0.49)	0.011

Change in ACR														
**≤50 years**	0.11 (-0.16-0.39)	0.421	0.03 (-0.10-0.16)	0.661	0.79 (0.06-1.53)	0.034	-0.22 (-0.65-0.20)	0.302	-0.22 (-1.62-1.18)	0.756	0.06 (-0.34-0.45)	0.767	-0.13 (-0.68-0.41)	0.635
**>50 years**	0.81 (-0.41-2.02)	0.194	0.12 (-0.67-0.92)	0.757	1.11 (-3.82-6.05)	0.658	0.63 (-0.25-1.51)	0.160	0.37 (-0.24-0.98)	0.237	-0.18 (-1.26-0.91)	0.747	2.60 (-4.90-10.1)	0.497

eGFR = estimated glomerular filtration rate, ACR = Albumin to creatinine ratio, SA Surinamese = South Asian Surinamese; Afr. Surinamese = African Surinamese

Table S9: Sensitivity analysis of association between sex and secondary outcomes CKD incidence and progression in the total population (ACR Subset)

	**Overall**		**Dutch**		**SA Surinamese**		**Afr. Surinamese**		**Ghanaian**		**Turkish**		**Moroccan**	
	OR [95% CI]	p-value	OR [95% CI]	p-value	OR [95% CI]	p-value	OR [95% CI]	p-value	OR [95% CI]	p-value	OR [95% CI]	p-value	OR [95% CI]	p-value
**CKD Incidence**														
**Model 1**	1.0 (0.8-1.2)	0.859	1.3 (0.8-2.1)	0.236	1.5 (1.0-2.2)	0.057	0.6 (0.4-1.0)	0.027	1.3 (0.8-2.2)	0.374	0.7 (0.3-1.6)	0.391	0.8 (0.4-1.5)	0.455
**Model 2**	1.0 (0.8-1.2)	0.919	1.1 (0.7-1.8)	0.582	1.7 (1.1-2.6)	0.011	0.6 (0.4-0.9)	0.024	1.2 (0.7-2.2)	0.466	0.8 (0.3-1.9)	0.595	0.8 (0.4-1.6)	0.586
**CKD Progression**														
**Model 1**	1.8 (1.2-2.7)	0.009	1.3 (0.3-4.9)	0.738	2.0 (0.9-4.6)	0.088	1.4 (0.5-3.7)	0.493	2.0 (0.5-7.6)	0.337	0.6 (0.1-3.4)	0.542	1.5 (0.4-6.4)	0.545
**Model 2**	1.7 (1.1-	0.010	0.8 (0.2-	0.801	2.0 (0.9-4.6)	0.084	1.6 (0.6-4.3)	0.372	1.8 (0.4-7.7)	0.418	1.0 (0.1-	0.989	1.6 (0.4-6.9)	0.498

	2.7)	3.5)	8.2)

*Defined as eGFR <60 mL/min/1.73 m2 and/or ACR of > 3 mg/mmol CKD Incidence analyses includes those without CKD at baseline; CKD progression analyses include those with CKD at baseline; Model 1: Adjusted for Baseline kidney function values & Follow up duration; Model 2: Model 1 + age, education & ethnicity; SA Surinamese = South Asian Surinamese; Afr. Surinamese = African Surinamese CKD = chronic kidney disease

Table S10: Sex differences in change in eGFR over time – Mediation analyses using "Mediation" package in r[1]

		ACME (95% CI)	p-value	ADE (95% CI)	p-value	Total effect (95% CI)	p-value	Proportion of mediation explained	p-value
**Overall**	Obesity	0.06 (0.01-0.12)	0.022	-0.08 (-0.42-0.28)	0.682	-0.02 (-0.36-0.34)	0.934	-0.10 (-5.03-4.74)	0.940
	Alcohol Consumption	-0.03 (-0.05- -0.01)	0.002	0.02 (-0.36-0.29)	0.914	-0.05 (-0.39-0.26)	0.784	0.10 (-2.31-3.00)	0.782
	Physical Activity	0.01 (-0.01-0.04)	0.350	-0.05 (-0.39-0.30)	0.810	-0.04 (-0.38-0.30)	0.860	-0.01 (-0.97-1.13)	0.900
	Smoking	-0.02 (-0.07-0.02)	0.320	0.001 (-0.37-0.37)	0.980	-0.02 (-0.38-0.34)	0.890	0.03 (-2.08-1.96)	0.920
	Hypertension	-0.09 (-0.13- -0.05)	0.000	0.05 (-0.30-0.41)	0.780	-0.04 (-0.39-0.32)	0.800	0.32 (-7.26-6.38)	0.800
	Diabetes	-0.02 (0.04-0.00)	0.040	-0.02 (-0.35-0.31)	0.870	-0.03 (-0.36-0.29)	0.820	0.04 (-1.07-0.99)	0.830
	CVD	-0.01 (-0.02-0.01)	0.450	-0.04 (-0.39-0.31)	0.830	-0.04 (-0.41-0.31)	0.810	-0.01 (-0.63-0.53)	0.880
**Dutch**	Obesity	0.01 (-0.05-0.01)	0.482	1.18 (0.56-1.77)	0.000	1.17 (0.55-1.76)	0.002	-0.01 (-0.4-0.01)	0.482
	Alcohol Consumption	-0.08 (-0.18-0.01)	0.072	1.26 (0.60-1.87)	0.000	1.17 (0.50-1.77)	0.000	-0.07 (-0.23-0.017	0.072
	Physical Activity	0.01 (-0.07-0.03)	0.530	1.21 (0.58-1.85)	0.000	1.20 (0.56-1.83)	0.000	-0.01 (-0.06-0.03)	0.530
	Smoking	-0.01 (0.04-0.02)	0.480	1.18 (0.54-1.81)	0.000	1.17 (0.53-1.80)	0.000	0.007 (-0.05-0.02)	0.480
	Hypertension	-0.15 (-0.28- -0.04)	0.010	1.32 (0.68-1.93)	0.000	1.17 (0.52-1.77)	0.002	-0.13 (-0.36- -0.03)	0.012
	Diabetes	-0.03 (-0.09-0.02)	0.290	1.21 (0.57-1.81)	0.000	1.18 (0.55-1.79)	0.000	-0.02 (-0.09-0.02)	0.290
	CVD	-0.02 (-0.07-0.01)	0.220	1.19 (0.56-1.81)	0.000	1.17 (0.53-1.79)	0.000	-0.02 (-0.07-0.01)	0.210
**SA Surinamese**	Obesity	0.15 (0.001-0.33)	0.046	-1.11 (-2.14- -0.04)	0.044	-0.96 (-1.98-0.12)	0.076	-0.14 (-1.37-0.50)	0.122

	Alcohol Consumption	0.04 (-0.04-0.15)	0.330	-1.01 (-2.1- -0.05)	0.034	-0.96 (-2.00- -0.00)	0.050	-0.04 (-0.41-0.13)	0.368
	Physical Activity	0.05 (-0.04-0.17)	0.330	-1.06 (-2.09-0.06)	0.034	-1.01 (-2.03- -0.03)	0.044	-0.04 (-0.51-0.19)	0.370
	Smoking	0.06 (-0.15-0.26)	0.546	-0.99 (-1.93-0.03)	0.044	-0.93 (-1.87-0.05)	0.066	-0.05 (-0.84-0.43)	0.592
	Hypertension	-0.09 (-0.23-0.02)	0.130	-0.90 (-1.89-0.11)	0.074	-0.99 (-1.99- -0.03)	0.054	0.08 (-0.13-0.54)	0.180
	Diabetes	-0.05 (-0.20-0.08)	0.432	-0.96 (-1.96-0.07)	0.064	-1.02 (-2.01-0.02)	0.058	0.05 (-0.33-0.34)	0.494
	CVD	-0.06 (-0.20-0.02)	0.142	-0.98 (-1.94-0.05)	0.064	-0.98 (-1.93-0.05)	0.064	0.05 (-0.12-0.48)	0.198
**Afr. Surinamese**	Obesity	0.20 (-0.01-0.43)	0.044	0.83 (-0.08-1.72)	0.076	1.03 (0.17-1.91)	0.008	0.19 (-0.002-1.27)	0.052
	Alcohol Consumption	0.05 (-0.01-0.15)	0.098	0.94 (0.12-1.80)	0.026	0.99 (0.17-1.87)	0.018	0.05 (-0.01-0.31)	0.108
	Physical Activity	0.02 (-0.12-0.07)	0.730	1.03 (0.16-1.90)	0.022	1.02 (0.18-1.86)	0.016	-0.01 (-0.18-0.10)	0.738
	Smoking	0.02 (-0.16-0.19)	0.842	0.98 (0.12-1.77)	0.034	0.98 (0.12-1.77)	0.022	0.02 (-0.27-0.39)	0.836
	Hypertension	0.005 (-0.07-0.09)	0.910	1.03 (0.14-1.89)	0.026	1.03 (0.13-1.88)	0.026	0.003 (-0.14-0.11)	0.976
	Diabetes	0.02 (-0.05-0.09)	0.610	1.00 (0.11-1.86)	0.030	1.02 (0.10-1.86´7)	0.024	0.01 (-0.08-0.14)	0.636
	CVD	0.05 (-0.03-0.14)	0.238	0.96 (0.16-1.83)	0.012	1.01 (0.20-1.88)	0.008	0.04 (-0.04-0.22)	0.228
**Ghanaian**	Obesity	-0.24 (-0.67-0.12)	0.200	-0.33 (-1.86-1.12)	0.690	-0.57 (-2.01-0.86)	0.420	0.20 (-4.17-4.57)	0.520
	Alcohol Consumption	-0.02 (-0.14-0.08)	0.620	-0.69 (-2.05-0.71)	0.340	-0.71 (-2.08-0.67)	0.330	0.01 (-0.55-0.63)	0.760
	Physical Activity	0.02 (-0.18-0.22)	0.830	-0.62 (-2.03-0.74)	0.450	-0.60 (-1.95-0.75)	0.450	-0.02 (-1.20-1.10)	0.850
	Smoking	0.10 (-0.16-0.40)	0.460	-0.78 (-2.18-0.62)	0.290	-0.68 (-2.04-0.69)	0.350	-0.07 (-2.18-2.06)	0.660
	Hypertension	0.01 (-0.07-0.12)	0.750	-0.63 (-1.95-0.75)	0.360	-0.63 (-1.94-0.78)	0.370	-0.006 (-0.56-0.37)	0.840
	Diabetes	0.01 (-0.11-0.14)	0.930	-0.67 (-2.13-0.75)	0.350	-0.67 (-2.13-0.73)	0.360	-0.001 (-0.49-0.45)	0.970
	CVD	0.005 (-0.10-0.07)	0.900	-0.64 (-1.92-0.80)	0.340	-0.64 (-1.94-0.78)	0.330	0.001 (-0.29-0.31)	0.940

**Turkish**	Obesity	-0.03 (-0.12-0.03)	0.420	-0.71 (-1.69-0.27)	0.160	-0.73 (-1.70-0.22)	0.220	0.02 (-0.26-0.36)	0.530
	Alcohol Consumption	-0.02 (-0.11-0.05)	0.590	-0.63 (-1.49-0.30)	0.170	-0.65 (-1.50-0.29)	0.160	0.01 (-0.22-0.55)	0.650
	Physical Activity	-0.01 (-0.13-0.11)	0.870	-0.73 (-1.67-0.23)	0.140	-0.74 (-1.67-0.20)	0.140	0.005 (-0.54-0.37)	0.930
	Smoking	-0.003 (-0.09-0.08)	0.960	-0.63 (-1.59-0.34)	0.180	-0.63 (-1.60-0.33)	0.190	0.001 (-0.32-0.42)	0.970
	Hypertension	-0.03 (-0.18-0.12)	0.730	-0.68 (-1.61-0.26)	0.140	-0.71 (-1.68-0.18)	0.120	0.03 (-0.78-0.63)	0.790
	Diabetes	-0.03 (-0.12-0.04)	0.450	-0.73 (-1.63-0.04)	0.120	-0.75 (-1.67-0.16)	0.110	0.02 (-0.17-0.40)	0.500
	CVD	0.01 (-0.09-0.06)	0.700	-0.72 (-1.61-0.17)	0.120	-0.73 (-1.64-0.15)	0.120	0.01 (-0.23-0.30)	0.730
**Moroccan**	Obesity	-0.02 (-0.15-0.12)	0.810	-1.79 (-2.72- -0.84)	0.000	-1.81 (-2.74- -0.86)	0.002	0.01 (-0.07-0.10)	0.810
	Alcohol Consumption	0.01 (-0.04-0.07)	0.690	-1.79 (-2.7- -0.07)	0.000	-1.78 (-2.72- -0.85)	0.000	-0.004 (-0.05-0.03)	0.690
	Physical Activity	-0.03 (-0.14-0.06)	0.550	-1.77 (-2.66- -0.88)	0.000	-1.80 (-2.70- -0.91)	0.000	0.02 (-0.04-0.09)	0.500
	Smoking	0.14 (-0.02-0.33)	0.090	-1.95 (-2.90- -1.09)	0.000	-1.81 (-2.69- -0.96)	0.000	-0.08 (-0.24-0.02)	0.130
	Hypertension	-0.09 (-0.21- -0.01)	0.018	-1.71 (-2.65- -0.72)	0.002	-1.80 (-2.73- -0.86)	0.000	0.05 (0.01-0.16)	0.020
	Diabetes	0.01 (-0.04-0.06)	0.780	-1.84 (-2.75- -0.90)	0.000	-1.83 (-2.75-0.90)	0.000	-0.002 (-0.04-0.02)	0.790
	CVD	.	1.0	.	0.000	.	0.000	.	1.0

ACME=Average Causal Mediation Effect (Indirect effect); ADE=Average Direct Effect; CVD= Cardiovascular disease; 95% CI= 95% Confidence Interval; SA Surinamese = South Asian Surinamese; Afr. Surinamese = African Surinamese, [1] Tingley D YT, Hirose K, Keele L, Imai K. mediation: R Package for Causal Mediation Analysis. Journal of Statistical Software; 2014. p. 1-38.

Table S11: Sex differences in change in eGFR over time – Mediation Analyses according to Baron and Kenny

		Mediator ~ change in eGFR		Sex -> Mediator		Change eGFR ~ Sex adjusted Model 2+ Mediator	
		β [95% CI]	p-value	OR [95% CI]	p-value	β [95% CI]	p-value
**Overall**	Obesity	-0.5 (-1.0- -0.1)	0.020	0.5 [0.4-0.5]	0.001	0.42 (0.1-0.8)	0.022
	Alcohol Consumption	1.4 (0.7-2.1)	0.000	0.74 (0.6-0.9)	0.000	-0.42 (-0.7- -0.1)	0.005
	Physical Activity	0.17 (-0.2-0.5)	0.354	1.3 (1.2-1.4)	0.000	0.05 (-0.05-0.2)	0.359
	Smoking	-0.21 (-0.6-0.2)	0.343	1.9 (1.7-2.1)	0.000	-0.14 (-0.4-0.2)	0.344
	Hypertension	-1.2 (-1.6- -0.8)	0.000	1.5 (1.4-1.6)	0.000	-0.5 (-0.7- -0.3)	0.000
	Diabetes	-0.8 (-1.5- -0.2)	0.010	1.3 (1.1-1.5)	0.001	-0.2 (-0.4- -0.1)	0.039
	CVD	-0.8 (-1.4- -0.3)	0.001	1.1 (0.9-1.2)	0.332	-0.1 (-0.2-0.1)	0.353
**Dutch**	Obesity	-1.1 (-2.2-0.0)	0.056	1.1 (0.9-1.5)	0.378	-0.13 (-0.4-0.2)	0.422
	Alcohol Consumption	1.4 (0.7-2.1)	0.000	0.5 (0.3-0.6)	0.000	-1.1 (-1.7- -0.5)	0.002
	Physical Activity	0.2 (-0.5-0.9)	0.552	0.7 (0.6-0.8)	0.001	0.1 (-0.3-0.2)	0.557
	Smoking	-0.6 (-1.4-0.1)	0.105	1.1 (0.9-1.3)	0.329	-0.1 (-0.2-0.1)	0.403
	Hypertension	-1.0 (-1.8- -0.3)	0.009	2.6 (2.1-3.1)	0.000	-1.0 (-1.8- -0.2)	0.012
	Diabetes	-1.2 (-3.0-0.6)	0.208	2.1 (1.4-3.3)	0.001	-0.9 (-2.3-0.6)	0.239
	CVD	-0.8 (-2.1-0.4)	0.183	1.5 (1.1-2.0)	0.007	-0.3 (-0.8-0.2)	0.233
**SA Surinamese**	Obesity	-1.5 (-2.8 - -0.2)	0.023	0.5 (0.3-0.6)	0.000	1.2 (0.1-2.2)	0.037
	Alcohol Consumption	1.4 (0.7-2.1)	0.000	2.5 (1.5-4.1)	0.001	1.3 (0.3-2.2)	0.001
	Physical Activity	0.5 (-0.5-1.5)	0.306	1.5 (1.3-1.9)	0.000	0.2 (-0.2-0.7)	0.320
	Smoking	0.4 (-0.8-1.6)	0.552	2.6 (2.0-3.2)	0.000	0.3 (-0.8-1.5)	0.553
	Hypertension	-0.9 (-2.0-0.3)	0.133	1.7 (1.4-2.2)	0.000	-0.5 (-1.1-0.2)	0.153

	Diabetes	-0.7 (-2.1-0.7)	0.326	1.9 (1.4-2.5)	0.000	-0.4 (-1.3-0.5)	0.337
	CVD	-1.2 (-2.5-0.1)	0.069	1.5 (1.1-1.9)	0.003	-0.5 (-1.0-0.1)	0.122
**Afr. Surinamese**	Obesity	-0.9 (-1.9-0.05)	0.006	0.3 (0.2-0.4)	0.000	1.2 (-0.1-2.4)	0.067
	Alcohol Consumption	2.2 (0.1-4.3)	0.037	1.8 (1.1-2.7)	0.012	1.2 (-0.3-2.7)	0.110
	Physical Activity	-0.2 (-1.0-0.7)	0.714	1.6 (1.3-1.9)	0.000	-0.1 (-0.5-0.3)	0.715
	Smoking	0.1 (-0.8-1.1)	0.807	2.5 (2.0-3.0)	0.000	0.1 (-0.8-1.0)	0.807
	Hypertension	-1.5 (-2.5- -0.6)	0.001	1.0 (0.8-1.2)	0.927	0.0 (-0.3-0.3)	0.927
	Diabetes	-1.9 (-3.4- -0.6)	0.006	0.9 (0.6-1.2)	0.496	0.2 (-0.4-0.8)	0.509
	CVD	-2.0 (-3.2- -0.8)	0.001	1.5 (1.1-1.9)	0.003	-0.8 (-1/4- -0.1)	0.028
**Ghanaian**	Obesity	0.8 (-0.5-2.2)	0.229	0.3 (0.2-0.4)	0.000	-1.1 (-2.8-0.8)	0.232
	Alcohol Consumption	1.0 (-4.2-6.1)	0.709	0.1 (0.0-0.1)	0.047	-2.1 (-13.3-9.1)	0.714
	Physical Activity	0.1 (-1.1-1.3)	0.855	1.9 (1.4-2.6)	0.000	0.1 (-0.7-0.9)	0.855
	Smoking	1.2 (-1.7-4.0)	0.424	6.2 (2.7-13.9)	0.000	2.1 (-3.1-7.3)	0.432
	Hypertension	0.8 (-0.5-2.1)	0.249	1.1 (0.8-1.5)	0.613	0.1 (-0.2-0.3)	0.643
	Diabetes	1.7 (-0.3-3.7)	0.097	1.0 (0.6-1.7)	0.906	0.1 (-0.8-0.9)	0.907
	CVD	0.231(-1.5-2.0)	0.795	0.8 (0.5-1.2)	0.330	-0.1 (-0.5-0.4)	0.801
**Turkish**	Obesity	0.6 (-0.4-1.7)	0.266	0.8 (0.5-1.1)	0.116	-0.2 (-0.5-0.2)	0.364
	Alcohol Consumption	-0.8 (-3.7-2.1)	0.588	2.2 (0.9-5.0)	0.061	-0.6 (-3.0-1.8)	0.603
	Physical Activity	-0.1 (-0.9-0.9)	0.928	1.6 (1.2-2.1)	0.001	0.0 (-0.4-0.4)	0.928
	Smoking	-0.0 (-1.0-1.0)	0.976	1.3 (1.0-1.8)	0.036	0.0 (-0.3-0.3)	0.976
	Hypertension	-0.2 (-1.4-1.0)	0.697	2.2 (1.6-3.1)	0.000	-0.2 (-1.1-0.8)	0.698
	Diabetes	-0.8 (-2.6-1.0)	0.392	1.7 (1.0-2.8)	0.037	-0.4 (-1.5-0.6)	0.429

	CVD	1.0 (0.2-2.2)	0.113	0.9 (0.6-1.3)	0.604	-0.1 (-0.4-0.3)	0.622
**Moroccan**	Obesity	0.1 (-0.8-1.1)	0.796	0.4 (0.3-0.5)	0.000	-0.1 (-0.9-0.7)	0.796
	Alcohol Consumption	0.7 (-2.9-4.3)	0.706	3.4 (1.2-10.1)	0.022	0.9 (-3.7-5.4)	0.709
	Physical Activity	-0.3 (-1.1-0.5)	0.472	1.6 (1.3-2.0)	0.000	-0.1 (-0.5-0.3)	0.477
	Smoking	0.9 (-0.4-2.3)	0.152	5.7 (3.8-8.5)	0.000	1.7 (-0.6-4.0)	0.157
	Hypertension	-1.6 (-2.6- -0.6)	0.002	1.5 (1.2-2.1)	0.002	-0.7 (-1.3- -0.1)	0.032
	Diabetes	1.1 (-0.2-2.4)	0.103	1.1 (0.7-1.5)	0.746	0.1 (-0.4-0.5)	0.751
	CVD	0.0 (-1.1-1.1)	0.991	0.9 (0.7-1.2)	0.422	0.0 (-0.1-0.1)	0.991

Model 2: Adjusted for Baseline kidney function estimates, follow up duration, age, ethnicity, and education; CVD = Cardiovascular Disease; eGFR = estimated Glomerular Filtration Rate; SA Surinamese = South Asian Surinamese; Afr. Surinamese = African Surinamese

Table S12: Mediation of association between sex and CKD Incidence over time in the total population (ACR Subset)

		ACME (95% CI)	p-value	ADE (95% CI)	p-value	Total effect (95% CI)	p-value	Proportion of mediation explained	p-value
**Overall**	Obesity	-0.003 (-0.004-0.00)	0.006	0.004 (-0.007-0.02)	0.454	-0.001 (-0.01-0.01)	0.804	0.31 (-10.42-6.63)	0.806
	Alcohol Consumption	.	.	.	.	.	.	.	.
	Physical Activity	-0.0004 (-0.001-0.00)	0.250	-0.0003 (-0.01-0.01)	0.930	-0.0001 (-0.01-0.01)	0.960	-0.006 (-1.39-1.69)	0.970
	Smoking	0.002 (-0.0003-0.00)	0.014	-0.0007 (-0.01-0.01)	0.884	0.001 (-0.01-0.01)	0.850	0.17 (-7.39-6.22)	0.852
	Hypertension	**0.003 (0.002-0.00)**	**0.000**	-0.002 (-0.01-0.01)	0.780	-0.001 (-0.01-0.01)	0.810	-0.33 (-7.58-8.41)	0.810
	Diabetes	**0.001 (0.0002-0.00)**	**0.016**	-0.0004 (-0.011-0.01)	0.942	-0.001 (-0.01-0.01)	0.868	-0.11 (-3.31-3.03)	0.868
	CVD	0.0003 (-0.0002-0.00)	0.260	-0.0002 (-0.01-0.01)	0.970	-0.0001 (-0.01-0.01)	0.970	-0.002 (-1.11-0.78)	0.970
**Dutch**	Obesity	0.0004 (-0.001-0.00)	0.520	-0.004 (-0.01-0.02)	0.620	-0.004 (-0.01-0.02)	0.580	-0.02 (-0.75-0.89)	0.770
	Alcohol Consumption	-0.0003 (-0.003-0.00)	0.880	-0.004 (-0.01-0.02)	0.610	0.004 (-0.01-0.02)	0.630	0.004 (-1.56-1.77)	0.980
	Physical Activity	-0.0002 (-0.002-0.00)	0.780	0.004 (-0.01-0.02)	0.900	-0.004 (-0.01-0.02)	0.660	0.01 (-0.92-1.02)	0.900
	Smoking	0.0002 (-0.001-0.00)	0.770	0.004 (-0.01-0.02)	0.630	0.004 (-0.01-0.02)	0.630	0.004 (-0.62-0.50)	0.900
	Hypertension	0.004 (0.0003-0.01)	0.028	-0.001 (-0.01-0.02)	0.874	-0.005 (-0.01-0.02)	0.538	-0.33 (-8.40-5.47)	0.538
	Diabetes	0.001 (-0.001-0.00)	0.380	-0.003 (-0.01-0.02)	0.670	-0.004 (0.01-0.02)	0.580	-0.03 (-1.12-1.56)	0.760
	CVD	0.0002 (-0.001-0.00)	0.790	-0.004 (-0.01-0.02)	0.610	0.005 (-0.01-0.02)	0.600	-0.002 (-0.52-0.54)	0.960
**SA**	Obesity	-0.005 (-0.01-	0.104	0.04 (0.01-0.07)	0.008	0.04 (0.01-0.07)	0.012	0.12 (-0.58-0.20)	0.104

Surinamese		0.00)						
	Alcohol Consumption	0.002 (-0.008-0.01)	0.310	0.04 (0.01-0.07)	0.026	0.04 (0.01-0.07)	0.018	0.03 (-0.28-0.26)	0.324
	Physical Activity	-0.002 (-0.01-0.00)	0.204	0.04 (0.01-0.07)	0.002	0.04 (0.01-0.07)	0.008	-0.05 (-0.33-0.04)	0.212
	Smoking	0.002 (-0.003-0.01)	0.516	0.04 (0.01-0.07)	0.014	0.04 (0.01-0.07)	0.010	0.05 (-0.13-0.31)	0.518
	Hypertension	0.009 (0.003-0.02)	0.000	0.03 (0.001-0.06)	0.044	0.04 (0.01-0.07)	0.012	0.22 (0.07-0.74)	0.012
	Diabetes	0.003 (-0.0004-0.01)	0.098	0.04 (0.01-0.07)	0.010	0.04 (0.01-0.08)	0.006	0.06 (-0.01-0.23)	0.100
	CVD	0.0004 (-0.002-0.00)	0.800	0.04 (0.01-0.07)	0.012	0.04 (0.01-0.07)	0.008	0.01 (-0.09-0.14)	0.800
**Afr. Surinamese**	Obesity	-0.006 (-0.01-0.00)	0.146	-0.03 (-0.05-0.00)	0.082	-0.03 (-0.06- -0.00)	0.030	0.17 (-0.14-1.04)	0.168
	Alcohol Consumption	0.0005 (-0.001-0.00)	0.554	-0.03 (-0.06- -0.01)	0.024	-0.03 (-0.06- -0.01)	0.028	-0.008 (-0.13-0.04)	0.570
	Physical Activity	0.001 (-0.003-0.00)	0.684	-0.03 (-0.06- -0.00)	0.024	-0.03 (-0.06- -0.00)	0.030	-0.02 (-0.30-0.13)	0.702
	Smoking	0.004 (-0.002-0.01)	0.168	-0.04 (-0.06- -0.01)	0.016	-0.03 (-0.06- -0.01)	0.022	-0.12 (-0.65-0.08)	0.186
	Hypertension	-0.001 (-0.004-0.00)	0.490	-0.03 (-0.06- -0.00)	0.046	-0.03 (-0.06- -0.00)	0.044	0.03 (-0.12-0.23)	0.498
	Diabetes	-0.002 (-0.006-0.00)	0.292	-0.03 (-0.06- -0.00)	0.040	-0.03 (-0.06- -0.01)	0.038	0.04 (-0.12-0.30)	0.318
	CVD	-0.001 (-0.003-0.00)	0.386	-0.03 (-0.06-0.00)	0.022	-0.03 (-0.06- -0.01)	0.016	0.02 (-0.04-0.19)	0.398
**Ghanaian**	Obesity	-0.005 (-0.02-0.01)	0.522	0.02 (-0.02-0.06)	0.320	0.02 (-0.02-0.05)	0.430	-0.12 (-3.69-3.49)	0.740
	Alcohol Consumption	0.003 (-0.0002-0.01)	0.056	0.01 (-0.03-0.05)	0.606	0.01 (-0.03-0.05)	0.512	0.09 (-1.56-1.58)	0.528
	Physical Activity	0.001 (-0.006-	0.760	0.01 (-0.03-0.06)	0.460	0.02 (-0.0120.06)	0.410	0.02 (-1.43-1.83)	0.890

		0.01)							
	Smoking	-0.002 (-0.01-0.01)	0.810	0.02 (-0.02-0.06)	0.380	0.02 (-0.02-0.06)	0.390	-0.02 (-1.61-1.13)	0.920
	Hypertension	0.0003 (-0.002-0.00)	0.740	0.02 (-0.03-0.06)	0.460	0.02 (-0.03-0.06)	0.450	0.008 (-0.55-0.68)	0.820
	Diabetes	-0.0001 (-0.006-0.01)	0.980	0.02 (-0.02-0.06)	0.420	0.02 (-0.02-0.05)	0.410	-0.008 (-1.17-1.61)	0.920
	CVD	-0.002 (-0.01-0.00)	0.480	0.02 (-0.02-0.05)	0.390	0.02 (-0.004-0.05)	0.390	-0.03 (-2.01-1.58)	0.770
**Turkish**	Obesity	.	.	.	.	.	.	.	.
	Alcohol Consumption	-0.002 (-0.002-0.01)	0.570	-0.01 (-0.04-0.03)	0.570	-0.007 (-0.04-0.03)	0.620	-0.016 (-1.26-1.37)	0.740
	Physical Activity	0.001 (-0.002-0.00)	0.710	-0.008 (-0.04-0.02)	0.590	-0.009 (-0.04-0.02)	0.570	-0.03 (-1.24-1.27)	0.800
	Smoking	0.0006 (-0.002-0.00)	0.710	-0.01 (-0.04-0.03)	0.550	-0.01 (-0.04-0.03)	0.560	-0.005 (-0.89-0.90)	0.890
	Hypertension	-0.002 (-0.003-0.01)	0.450	-0.01 (-0.04-0.03)	0.550	-0.007 (-0.04-0.03)	0.630	0.04 (-2.77-1.88)	0.810
	Diabetes	0.004 (-0.002-0.01)	0.200	-0.01 (-0.05-0.02)	0.490	-0.008 (-0.04-0.03)	0.620	-0.08 (-3.28-2.43)	0.750
	CVD	.	.	.	.	.	.	.	.
**Moroccan**	Obesity	-0.003 (-0.01-0.00)	0.510	-0.004 (-0.03-0.02)	0.780	-0.007 (-0.04-0.02)	0.620	0.07 (-3.31-7.04)	0.810
	Alcohol Consumption	-0.0006 (-0.002-0.00)	0.100	-0.007 (-0.03-0.02)	0.600	-0.007 (-0.03-0.02)	0.560	0.03 (-0.60-0.79)	0.630
	Physical Activity	-0.003 (-0.008-0.03)	0.780	-0.004 (-0.03-0.03)	0.780	-0.007 (-0.03-0.02)	0.660	0.10 (-3.35-2.38)	0.700
	Smoking	0.004 (-0.002-0.01)	0.330	-0.01 (-0.04-0.02)	0.440	-0.007 (0.03-0.02)	0.600	-0.15 (-4.98-4.79)	0.690
	Hypertension	0.0002 (-0.002-0.00)	0.820	-0.008 (-0.03-0.02)	0.590	-0.008 (-0.03-0.02)	0.610	-0.004 (-0.73-0.48)	0.890

	Diabetes	0.0002 (-0.001-0.00)	0.850	-0.007 (-0.03-0.02)	0.570	-0.007 (-0.03-0.02)	0.570	-0.0006 (-0.44-0.50)	0.950
	CVD	-0.0004 (-0.002-0.00)	0.640	-0.007 (-0.03-0.02)	0.620	-0.007 (-0.03-0.02)	0.600	0.006 (-0.87-0.50)	0.870

Measurements conducted in subgroup with available ACR samples , ACME=Average Causal Mediation Effect (Indirect effect); ADE=Average Direct Effect; CVD= Cardiovascular disease; 95% CI= 95% Confidence Interval; SA Surinamese = South Asian Surinamese; Afr. Surinamese = African Surinamese

Table S13: Sensitivity analysis of association between sex and CKD Progression over time in the total population (ACR Subset)

		ACME (95% CI)	p-value	ADE (95% CI)	p-value	Total effect (95% CI)	p-value	Proportion of mediation explained	p-value
**Overall**	Obesity	-0.002 (-0.004-0.00)	0.008	0.01 (0.004-0.02)	0.000	0.01 (0.002-0.01)	0.008	-0.28 (-0.88- -0.11)	0.008
	Alcohol Consumption	-0.0002 (-0.001-0.00)	0.620	0.05 (0.03-0.07)	0.000	0.05 (0.03-0.07)	0.000	-0.01 (-0.03-0.01)	0.620
	Physical Activity	-0.0001 (-0.001-0.00)	0.002	0.01 (0.001-0.00)	0.008	0.01 (0.001-0.01)	0.014	-0.08 (-0.51- -0.01)	0.016
	Smoking	0.003 (-0.0001-0.01)	0.068	0.05 (0.02-0.07)	0.000	0.05 (0.03-0.07)	0.000	0.05 (-0.002-0.15)	0.068
	Hypertension	0.001 (0.0003-0.00)	0.002	0.006 (0.001-0.01)	0.024	0.01 (0.002-0.01)	0.012	0.013 (0.04-0.48)	0.014
	Diabetes	0.002 (0.0002-0.00)	0.020	0.05 (0.02-0.07)	0.000	0.05 (0.02-0.07)	0.000	0.04 (0.004-0.10)	0.020
	CVD	0.001 (-0.0002-0.00)	0.300	0.01 (0.001-0.01)	0.010	0.01 (0.002-0.01)	0.010	0.02 (-0.03-0.011)	0.310
**Dutch**	Obesity	0.001 (-0.001-0.00)	0.560	0.03 (-0.01-0.07)	0.130	0.03 (-0.01-0.07)	0.120	0.01 (-0.19-0.22)	0.600
	Alcohol Consumption	-0.001 (-0.01-0.00)	0.240	-0.001 (-0.01-0.01)	0.860	-0.002 (-0.01-0.01)	0.690	0.09 (-3.15-3.45)	0.720
	Physical Activity	0.002 (-0.001-0.00)	0.620	-0.0004 (-0.01-0.01)	0.850	-0.001 (-0.01-0.01)	0.830	0.04 (-1.77-1.45)	0.740
	Smoking	0.00 (-0.001-0.00)	0.900	0.03 (-0.01-0.07)	0.140	0.03 (-0.01-0.07)	0.140	0.001 (-0.15-0.13)	0.910
	Hypertension	0.01 (-0.002-0.01)	0.170	0.03 (-0.01-0.07)	0.220	0.03 (-0.01-0.07)	0.140	0.15 (-1.87-1.77)	0.290
	Diabetes	0.0001 (-0.002-0.00)	0.170	-0.003 (-0.01-0.01)	0.540	-0.002 (-0.01-0.01)	0.670	-0.09 (-5.16-2.56)	0.710
	CVD	0.002 (0.00-0.01)	0.076	0.03 (-0.01-0.07)	0.166	0.03 (-0.01-0.07)	0.128	0.06 (-0.57-0.76)	0.760

**SA Surinamese**	Obesity	-0.01 (-0.01-0.00)	0.002	0.02 (0.003-0.04)	0.046	0.01 (-0.003-0.03)	0.116	-0.28 (-2.40-1.62)	0.118
	Alcohol Consumption	0.0004 (-0.001-0.00)	0.910	0.01 (-0.004-0.03)	0.110	0.01 (-0.004-0.04)	0.110	0.004 (-0.19-0.37)	0.900
	Physical Activity	-0.003 (-0.01-0.00)	0.018	0.02 (-0.002-0.04)	0.090	0.01 (-0.01-0.04)	0.182	-0.17 (-2.57-1.97)	0.196
	Smoking	0.001 (-0.001-0.01)	0.360	0.01 (-0.01-0.03)	0.170	0.01 (-0.003-0.04)	0.100	0.08 (-0.75-1.23)	0.440
	Hypertension	0.003 (-0.001-0.01)	0.004	0.01 (-0.01-0.03)	0.202	0.01 (-0.003-0.03)	0.108	0.18 (-0.43-1.79)	0.112
	Diabetes	0.001 (0.001-0.01)	0.000	0.01 (-0.01-0.03)	0.290	0.01 (-0.004-0.03)	0.100	0.36 (-1.06-2.73)	0.100
	CVD	0.001 (-0.001-0.00)	0.410	0.01 (-0.01-0.04)	0.170	0.01 (-0.004-0.04)	0.130	0.03 (-0.55-0.83)	0.520
**Afr. Surinamese**	Obesity	0.0002 (-0.004-0.00)	0.840	0.01 (-0.01-0.02)	0.420	0.01 (-0.01-0.02)	0.400	0.04 (-2.56-2.07)	0.870
	Alcohol Consumption	0.0002 (-0.001-0.00)	0.700	0.01 (-0.01-0.02)	0.360	0.01 (-0.01-0.02)	0.380	0.01 (-0.80-0.68)	0.870
	Physical Activity	0.01 (-0.001-0.01)	0.088	0.01 (-0.04-0.07)	0.608	0.02 (-0.03-0.07)	0.452	0.13 (-2.4-3.24)	0.500
	Smoking	0.0003 (-0.002-0.00)	0.880	0.01 (-0.01-0.02)	0.450	0.01 (-0.01-0.02)	0.440	0.02 (-1.62-1.47)	0.880
	Hypertension	-0.003 (0.003-000)	0.750	0.02 (-0.03-0.08)	0.420	0.02 (-0.03-0.08)	0.430	-0.002 (-0.36-0.30)	0.910
	Diabetes	-0.001 (-0.003-0.00)	0.510	0.01 (-0.01-0.02)	0.280	0.01 (-0.01-0.02)	0.320	-0.03 (-1.08-1.22)	0.700
	CVD	-0.001 (-0.001-0.00)	0.770	0.01 (-0.01-0.02)	0.350	0.01 (-0.01-0.02)	0.370	-0.001 (-0.50-0.62)	0.920
**Ghanaian**	Obesity	-0.003 (-0.02-0.01)	0.660	0.02 (-0.02-0.07)	0.380	0.01 (-0.03-0.07)	0.520	0.003 (-2.56-3.40)	0.990
	Alcohol	-0.003 (-0.02-	0.570	0.12 (0.04-0.19)	0.002	0.11 (0.04-0.19)	0.006	-0.02 (-0.19-0.07)	0.576

	Consumption	0.01)							
	Physical Activity	0.002 (-0.01 (0.00)	0.310	0.01 (0.03-0.07)	0.480	0.01 (0.03-0.07)	0.480	-0.07 (-2.21-1.28)	0.610
	Smoking	-0.002 (-0.01-0.00)	0.020	0.01 (-0.02-0.06)	0.488	0.01 (0.03-1.66)	0.558	-0.07 (-1.48-1.69)	0.562
	Hypertension	0.0002 (-0.004-0.00)	0.880	0.01 (0.03-0.07)	0.550	0.01 (0.03-0.07)	0.550	0.003 (-0.45-0.55)	0.920
	Diabetes	0.001 (-0.01-0.01)	0.794	0.12 (0.04-0.19)	0.002	0.12 (0.04-0.19)	0.002	-0.01 (-0.16-0.07)	0.796
	CVD	-0.001 (-0.01-0.00)	0.650	0.01 (0.03-0.06)	0.500	0.01 (0.03-0.06)	0.520	-0.007 (-0.07-0.50)	0.860
**Turkish**	Obesity	-0.0003 (-0.01-0.00)	0.870	0.06 (-0.01-0.13)	0.120	0.06 (-0.01-0.13)	0.130	-0.001 (-0.23-0.23)	0.890
	Alcohol Consumption	-0.002 (-0.002-0.01)	0.470	0.003 (-0.03-0.01)	0.810	0.001 (-0.03-0.02)	0.980	0.03 (-3.28-4.78)	0.830
	Physical Activity	-0.001 (-0.01-0.01)	0.560	-0.001 (-0.02-0.02)	0.990	0.001 (-0.02-0.01)	0.890	0.02 (-4.52-3.74)	0.940
	Smoking	0.001 (-0.001-0.01)	0.770	0.05 (-0.02-0.12)	0.180	0.05 (-0.02-0.12)	0.180	0.01 (-0.31-0.49)	0.800
	Hypertension	-0.01 (-0.03-0.00)	0.064	0.07 (-0.01-0.15)	0.078	0.06 (-0.02-0.14)	0.122	-0.16 (-1.57-0.82)	0.182
	Diabetes	0.001 (-0.002-0.01)	0.450	0.004 (-0.03-0.03)	0.990	0.002 (-0.02-0.03)	0.810	0.03 (-2.62-4.14)	0.830
	CVD	.	.	.	.	.	.	.	.
**Moroccan**	Obesity	-0.004 (-0.01-0.00)	0.150	0.008 (-0.01-0.03)	0.440	0.004 (-0.01-0.02)	0.620	-0.35 (-4.73-7.78)	0.540
	Alcohol Consumption	0.0002 (-0.001-0.00)	0.210	0.006 (-0.001-0.00)	0.470	0.005 (-0.01-0.02)	0.490	-0.01 (-0.43-0.37)	0.610
	Physical Activity	0.002 (-0.002-0.00)	0.880	0.005 (-0.01-0.03)	0.500	0.005 (-0.01-0.03)	0.470	0.01 (-1.02-1.13)	0.870

	Smoking	-0.002 (-0.005-0.00)	0.004	0.007 (-0.009-0.03)	0.394	0.005 (-0.01-0.03)	0.528	-0.16 (-2.75-2.33)	0.532
	Hypertension	-0.001 (-0.01-0.00)	0.420	0.002 (-0.02-0.02)	0.780	0.003 (-0.02-0.03)	0.780	-0.04 (-1.46-2.17)	0.760
	Diabetes	0.0002 (-0.001-0.00)	0.780	0.005 (-0.02-0.03)	0.610	0.005 (-0.01-0.03)	0.600	0.004 (-0.70-0.91)	0.910
	CVD	0.002 (-0.001-0.00)	0.790	0.005 (-0.01-0.03)	0.570	0.005 (-0.01-0.03)	0.540	0.002 (-0.86-0.78)	0.920

*Analyses conducted in subgroup with available ACR measurements at baseline; Adjusted for Baseline kidney function values, Follow up duration, Age, Education & Ethnicity (Model 2); ACR = Albumin to Creatinine Ratio; SA Surinamese = South Asian Surinamese; Afr. Surinamese = African Surinamese CVD = cardiovascular disease
